# Baseline demographics of a contemporary Belgian atrial fibrillation cohort included in a large randomised clinical trial on targeted education and integrated care (AF-EduCare/AF-EduApp study)

**DOI:** 10.3389/fcvm.2023.1186453

**Published:** 2023-06-02

**Authors:** Michiel Delesie, Lieselotte Knaepen, Paul Dendale, Johan Vijgen, Joris Ector, Lien Desteghe, Hein Heidbuchel

**Affiliations:** ^1^ Research Group Cardiovascular Diseases, University of Antwerp, Antwerp, Belgium; ^2^ Department of Cardiology, Heart Centre Hasselt, Jessa Hospital, Hasselt, Belgium; ^3^ Faculty of Medicine and Life Sciences, Hasselt University, Hasselt, Belgium; ^4^ Department of Cardiology, Antwerp University Hospital, Edegem, Belgium; ^5^ Department of Cardiology, University Hospital Gasthuisberg, Leuven, Belgium

**Keywords:** atrial fibrillation, integrated care, demographics, education, cardiovascular comorbidities

## Abstract

**Background:**

As the prevalence of atrial fibrillation (AF) increases worldwide and AF management becomes ever more diversified and personalised, insights into (regional) AF patient demographics and contemporary AF management are needed. This paper reports the current AF management and baseline demographics of a Belgian AF population recruited for a large multicenter integrated AF study (AF-EduCare/AF-EduApp study).

**Methods:**

We analyzed data from 1,979 AF patients, assessed between 2018 and 2021 for the AF-EduCare/AF-EduApp study. The trial randomised consecutive patients with AF (irrespective of AF history duration) into three educational intervention groups (in person-, online-, and application-based), compared with standard care. Baseline demographics of both the included and excluded/refused patients are reported.

**Results:**

The mean age of the trial population was 71.2 ± 9.1 years, with a mean CHA_2_DS_2_-VASc score of 3.4 ± 1.8. Of all screened patients, 42.4% were asymptomatic at presentation. Being overweight was the most common comorbidty, present in 68.9%, while 65.0% were diagnosed with hypertension. Anticoagulation therapy was prescribed in 90.9% of the total population and in 94.0% of the patients with an indication for thromboembolic prophylaxis. Of the 1,979 assessed AF patients, 1,232 (62.3%) were enrolled in the AF-EduCare/AF-EduApp study, with transportation problems (33.4%) as the main reason for refusal/non-inclusion. About half of the included patients were recruited at the cardiology ward (53.8%). AF was first diagnosed, paroxysmal, persistent and permanent in 13.9%, 47.4%, 22.8% and 11.3%, respectively. Patients who refused or were excluded were older (73.3 ± 9.2 vs. 69.8 ± 8.9 years, *p* < 0.001) and had more comorbidities (CHA_2_DS_2_-VASc 3.8 ± 1.8 vs. 3.1 ± 1.7, *p* < 0.001). The four AF-EduCare/AF-EduApp study groups were comparable across the vast majority of parameters.

**Conclusions:**

The population showed high use of anticoagulation therapy, in line with current guidelines. In contrast to other AF trials about integrated care, the AF-EduCare/AF-EduApp study managed to incorporate all types of AF patients, both out-patient and hospitalised, with very comparable patient demographics across all subgroups. The trial will analyze whether different approaches to patient education and integrated AF care have an impact on clinical outcomes.

**Clinical Trial Registration:**

https://clinicaltrials.gov/ct2/show/NCT03707873?term=af-educare&draw=2&rank=1, identifier: NCT03707873; https://clinicaltrials.gov/ct2/show/NCT03788044?term=af-eduapp&draw=2&rank=1, identifier: NCT03788044.

## 1. Introduction

Atrial Fibrillation (AF) is an emerging epidemic in Western countries and creates a high burden on patients, healthcare providers and healthcare systems (1). Currently, the lifetime risk for developing AF in adults above 55 years old is 37% and it is estimated that in 2060, AF will affect 17.9 million European citizens (i.e., 3.5% of the total population) (2, 3). This is due to ageing of the population and the increasing prevalence of modifiable AF risk factors such as hypertension, diabetes mellitus, obesity, heart failure and obstructive sleep apnea (OSA), which all contribute to the development and the progression of AF (4).

AF is related with several clinical outcomes like an overall 3.5-fold mortality risk, responsible for 20%–30% of all ischemic strokes, a 10%–40% annual hospitalisation rate, left ventricular dysfunction in 20%–30%, and impaired quality of life in more than 60% of AF patients (4).

AF care is multidimensional. This complexity requires great efforts of all health care providers and big investments from healthcare systems. Ideally, it requires a patient-centered, multidisciplinary, integrated and structured approach as proposed by the 2016 and 2020 European Society of Cardiology (ESC) guidelines. Moreover, the management of AF needs to be tailored to regional AF patient characteristics and health care realities (4, 5). The optimal determining components and global approach of such integrated care is not fully established and requires further study.

Our research group had shown before that short tailored education sessions based on patients' knowledge gaps assessed with the Jessa Atrial fibrillation Knowledge Questionnaire (JAKQ) significantly improved their knowledge both via in-person and online education (6, 7). Therefore, the innovative integrated care approach of the AF-EduCare/AF-EduApp studies (NCT03707873 & NCT03788044) is based on this type of (I) education combined with (II) systematic assessment of AF risk factors, (iii) patient involvement to improve self-care capabilities, improvement of adherence to oral anticoagulation (OAC) therapy and (iv) low-threshold accessibility for study patients to the care team in case of AF-related questions or problems. The main goal is to improve several clinical outcomes (8).

This paper describes the contemporary Belgian AF population of unselected consecutive AF patients, recruited for the trial from both outpatient clinics as hospitalisation wards. Moreover, we explore the uniformity of the baseline demographic data in the different AF-EduCare/AF-EduApp study groups.

## 2. Methods

The AF-EduCare study (ClinicalTrials.gov—NCT03707873) is an open, prospective, randomised clinical trial (RCT) conducted in three Belgian tertiary centers (Antwerp University Hospital, the Jessa Hospital in Hasselt and the University Hospitals Leuven) (8). A total of 1,038 AF patients were randomised to three study groups (in-person education—online education—standard care).

The AF-EduApp study (ClinicalTrials.gov—NCT03788044) evaluates an integrated care application (operating on a tablet or smartphone) for AF patients with the primary aim to improve adherence to non-vitamin K-antagonist oral anticoagulants (NOAC). This extra study arm was integrated in the AF-Educare study (and in its randomisation process) for which an additional 153 AF patients (on-treatment) were recruited at the Antwerp University Hospital and the Jessa Hospital in Hasselt.

The Ethics Committees of the participating centers approved both trials and its amendments Belgian study number B300201836720—Ethics committee approval n° 18/12/171). These studies are being conducted in compliance with the Declaration of Helsinki.

### 2.1. Study population and procedure

Patients with AF (diagnosed with a single-lead ECG recording of ≥30 s or a 12-lead ECG), hospitalised at the department of cardiology or who presented for an outpatient visit, were assessed for the AF-EduCare/AF-EduApp study. All types of AF patients were eligible (1) with a minimum age of 18 years, (2) AF or atrial flutter diagnosed with an electrocardiogram, and (3) capable of signing the informed consent. Exclusion criteria were (1) not able to speak and read Dutch, (2) cognitively impaired (e.g., severe dementia), (3) life expectancy estimated <1 year, (4) participation in another randomised clinical trial and (5) pregnant women. After enrolment and providing written consent, their clinical data and profile were registered in the electronic Case Report Form (eCRF) before the start of any intervention. AF patients who were not eligible or not willing to participate were also logged in the eCRF to avoid readdressing these candidates twice during the inclusion period. Only baseline demographic data of these patients were retrieved from the patients' hospital files.

### 2.2. Data

All collected data were stored in an encoded eCRF. Each participating center kept a separate list linking the eCRF study number with the study patient identification. This list was only accessible by the local investigators so that this information was maximally secured. Baseline data of all patients was defined as the data on the date of study inclusion or on the date of the study enrolment proposal for the excluded/refused patients.

## 3. Statistics

Data were analysed using IBM SPSS version 28.0. Variables were described as numbers and percentages or as mean ± standard deviation, as appropriate. Normal distribution was assumed as all study subgroups were large enough. For continuous variables, differences between two or more groups were compared using the independent T-test or one-way ANOVA analysis. The chi-squared test and Fisher's exact test were used for categorical variables, when appropriate. *P*-values < 0.05 were considered statistically significant. Of note, non-objectified parameters in the medical file of the excluded/refused patients were considered to be unknown and were left out in the analysis and comparisons.

## 4. Results

### 4.1. Enrolment

Enrolment of patients began in September 2018 and ended in March 2021. Due to the development and validation of the AF-EduApp, inclusions for this study started in October 2019. The anticipated 18 month inclusion period was expanded due to the COVID-19 outbreak in 2020, which abruptly interrupted patient recruitment in all centers.

**Figure 1:**
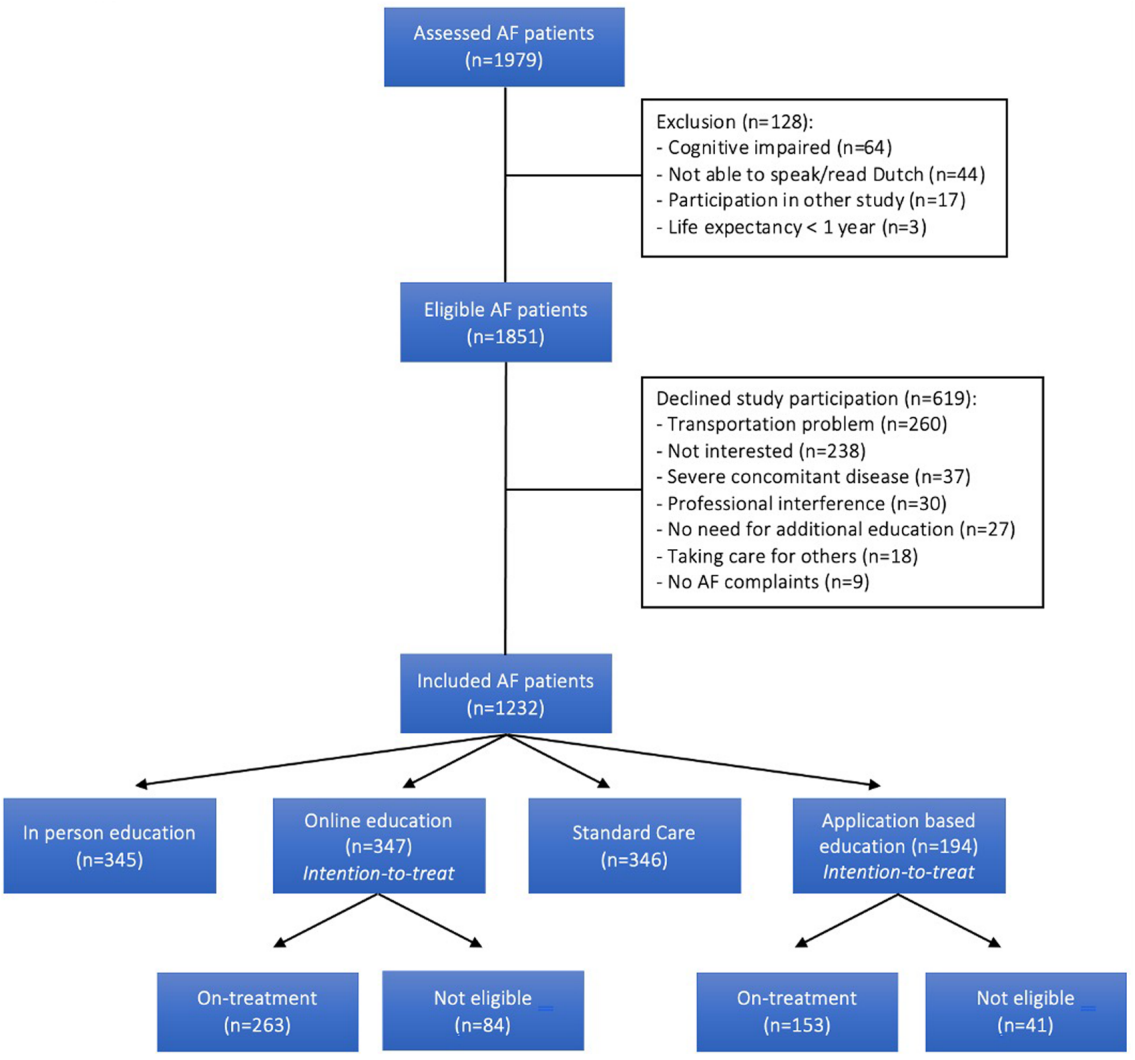
Enrolment.

A total of 1,979 AF patients were assessed for study participation (Figure 1). Of these, 128 (6.5%) were excluded, mainly due to cognitive impairment (50.0%). Of the resulting 1,851 eligible AF patients, 619 declined participation (33.4%) primarily because of transportation problems (e.g., distance to the hospital; depending on others; living abroad) and insufficient interest in the study (e.g., no time; only preferring follow-up by their treating physician) in 42.0% and 38.4% of patients, respectively. A total of 1,232 AF patients were eventually included of which 1,038 (84.3%) and 194 (15.7%) patients were enrolled in the AF-EduCare- and AF-EduApp trial, respectively. Of the patients randomised to the online- (*n* = 347) and application-based (*n* = 194) education groups, 75.8% (*n* = 263) and 78.8% (*n* = 153) respectively were eligible of using the online platform or using the in-house developed AF application (=on-treatment subgroups). Future outcome analysis of these two study arms will be performed as intention-to-treat.

### 4.2. AF patient demographics

Of the 1,979 AF patients, slightly more than half were approached at the cardiology ward (59.2%), of which 79.6% were hospitalised primarily related to their AF while 23.7% were cardiovascular unplanned admissions.

Table 1 presents the baseline characteristics of the entire patient cohort, i.e., the included, excluded and declined patients. Mean age was 71.2 ± 9.1 years, 65.3% were male and 98.9% were Caucasian. Almost half of the patients had paroxysmal AF (48.7%) and mean duration since AF diagnosis was 5.8 ± 6.9 years.

**Table 1:** Baseline characteristics of the assessed AF patients.

	Total AF population (*n* = 1,979)	Included (*n* = 1,232)	Excluded/not willing to participate (n = 747)	*P*-value
Enrolment at outpatient clinic, *n* (%)	807 (40.8)	569 (46.2)	238 (31.9)	**<0**.**001**
AF-related, *n* (%)	668 (82.8)	505 (88.8)	163 (68.5)	**<0**.**001**
Unplanned, *n* (%)	15 (1.9)	9 (1.6)	6 (2.5)	0.368
Enrolment at cardiology ward, *n* (%)	1,172 (59.2)	663 (53.8)	509 (68.1)	**<0**.**001**
AF-related, *n* (%)	933 (79.6)	587 (88.5)	346 (68.0)	**<0**.**001**
Unplanned, *n* (%)	278 (23.7)	133 (20.1)	145 (28.5)	**<0**.**001**
Treated by electrophysiologist	1,045 (52.8)	697 (56.6)	348 (46.6)	**<0**.**001**
Age (years), mean ± SD	71.2 ± 9.1	69.8 ± 8.9	73.3 ± 9.2	**<0**.**001**
Male, *n* (%)	1,292 (65.3)	851 (69.1)	441 (59.0)	**<0**.**001**
Belgian nationality, *n* (%)	1,916 (96.8)	1,201 (97.5)	715 (95.7)	**0**.**030**
Race, *n* (%)			*(n = 691)*	**0**.**006**
Caucasian, *n* (%)	1,903 (98.9)	1,225 (99.4)	678 (98.1)	
Other, *n* (%)	20 (1.0)	7 (0.6)	13 (1.9)	
BMI (kg/m^2^), mean ± SD	28.0 ± 5.1	27.9 ± 4.9	28.1 ± 5.4	0.653
			*(n = 737)*	
BMI categories, *n* (%)			*(n = 737)*	**0**.**022**
≤25 kg/m^2^	613 (31.1)	366 (29.7)	247 (33.5)	
25–30 kg/m^2^	766 (38.9)	508 (41.2)	258 (35.0)	
≥30 kg/m^2^	590 (30.0)	358 (29.1)	232 (31.5)	
Kind of AF, *n* (%)			*(n = 744)*	**0**.**005**
First diagnosed	245 (12.4)	171 (13.9)	74 (9.9)	
Paroxysmal AF	962 (48.7)	584 (47.4)	378 (50.8)	
Persistent AF	420 (21.3)	281 (22.8)	139 (18.7)	
Long-standing persistent AF	14 (0.7)	9 (0.7)	5 (0.7)	
Permanent AF	247 (12.5)	139 (11.3)	108 (14.5)	
Atrial flutter	88 (4.5)	48 (3.9)	40 (5.4)	
Time since AF diagnosis (years), mean ± SD	5.8 ± 6.9	5.8 ± 7.2	5.6 ± 6.4	0.424
Rhythm at baseline, *n* (%)				**<0**.**001**
Sinus rhythm	1,261 (64.0)	933 (75.7)	328 (44.4)	** **
AF/Atrial flutter	642 (32.6)	275 (22.3)	367 (49.7)	** **
Other rhythm	67 (3.4)	24 (1.9)	43 (5.8)	** **
CHA_2_DS_2_-VASc score, mean ± SD	3.4 ± 1.8	3.1 ± 1.7	3.8 ± 1.8	**<0**.**001**
CHA_2_DS_2_-VASc classification, n (%)				**<0**.**001**
CHA_2_DS_2_-VASc score 0 (m) or 1 (f)	124 (6.3)	88 (7.1)	36 (4.8)	** **
CHA_2_DS_2_-VASc score 1 (m) or 2 (f)	255 (12.9)	187 (15.2)	68 (9.1)	** **
CHA_2_DS_2_-VASc score ≥2 (m) or ≥3 (f)	1,600 (80.8)	957 (77.7)	643 (86.1)	** **
HAS-BLED score, mean ± SD	1.6 ± 0.9	1.5 ± 0.9	1.6 ± 1.0	**0**.**003**
HAS-BLED classification, *n* (%)				0.134
HAS-BLED 0–2	1,709 (86.4)	1,075 (87.3)	634 (84.9)	** **
HAS-BLED ≥ 3	270 (13.6)	157 (12.7)	113 (15.1)	** **
mEHRA, *n* (%)			*(n = 567)*	**0**.**046**
1	762 (42.4)	514 (41.7)	248 (43.7)	
2a	502 (27.9)	361 (29.3)	141 (24.9)	
2b	289 (16.1)	204 (16.6)	85 (15.0)	
3	219 (12.2)	139 (11.3)	80 (14.1)	
4	27 (1.5)	14 (1.1)	13 (2.3)	
Concomitant disease, *n* (%)				
(Coronary) artery disease	662 (33.5)	385 (31.3)	277 (37.1)	**0**.**008**
History of congestive heart failure	777 (39.3)	439 (35.6)	338 (45.2)	**<0**.**001**
NYHA class III/IV	198 (27.3)	91 (20.7)	107 (37.5)	**<0**.**001**
			* (n = 285)*	
Heart failure classification			*(n = 332)*	**0**.**013**
HFpEF	348 (45.1)	213 (48.5)	135 (40.6)	** **
HFmrEF	158 (20.5)	93 (21.2)	65 (19.4)	
HFrEF	268 (34.7)	133 (30.3)	135 (40.7)	
Thyroid disease				0.271
Hyperthyroidism	121 (6.1)	67 (5.4)	54 (7.2)	** **
Hypothyroidism	183 (9.2)	114 (9.3)	69 (9.2)	** **
Severe kidney dysfunction^a^	105 (5.3)	46 (3.7)	59 (7.9)	**<0**.**001**
COPD	143 (7.2)	81 (6.6)	62 (8.3)	0.151
Active malignancy	89 (4.5)	35 (2.8)	54 (7.2)	**<0**.**001**
Liver disease	23 (1.2)	9 (0.7)	14 (1.9)	**0**.**021**
Cardiovascular risk factors, *n* (%)				
Diabetes mellitus type I/II	411 (20.8)	220 (17.9)	191 (25.6)	**<0**.**001**
Hypertension	1,287 (65.0)	759 (61.6)	528 (70.7)	**<0**.**001**
Hypercholesterolemia	1,387 (70.3)	848 (69.1)	539 (72.4)	0.110
		*(n = 1,228)*	*(n = 744)*	
Current smoker	175 (9.1)	99 (8.0)	76 (11.0)	**0**.**033**
			*(n = 694)*	
Alcohol excess (≥8/week)	354 (22.3)	299 (24.3)	55 (15.5)	**<0**.**001**
			*(n = 355)*	
Documented diagnosis of OSA	214 (11.2)	152 (12.6)	62 (8.8)	**0**.**010**
		* (n = 1,209)*	*(n = 708)*	
Co-morbidities, *n* (%)				
Previous TIA	117 (5.9)	78 (6.3)	39 (5.2)	0.310
Previous ischaemic stroke	150 (7.6)	83 (6.7)	67 (9.0)	0.069
Previous hemorrhagic stroke	9 (0.5)	3 (0.2)	6 (0.8)	0.073
Other ischaemic thrombo-embolic events	13 (0.7)	9 (0.7)	4 (0.5)	0.603
History of pulmonary embolism	40 (2.0)	19 (1.5)	21 (2.8)	0.052
Bleeding history	57 (2.9)	26 (2.1)	31 (4.1)	**0**.**009**
Devices				0.139
PM	184 (9.3)	99 (8.0)	85 (11.4)	
ICD	95 (4.8)	58 (4.7)	37 (5.0)	
CRT-PM	32 (1.6)	18 (1.5)	14 (1.9)	
CRT-ICD	43 (2.2)	25 (2.0)	18 (2.4)	
Anticoagulation therapy, *n* (%)				0.237
NOAC	1,620 (81.9)	995 (80.8)	625 (83.7)	
Apixaban	540 (33.3)	316 (31.8)	224 (35.8)	0.256
Edoxaban	535 (33.0)	339 (34.1)	196 (31.4)	
Rivaroxaban	376 (23.2)	240 (24.1)	136 (21.8)	
Dabigatran	169 (10.4)	100 (10.1)	69 (11.0)	
VKA	159 (8.0)	105 (8.5)	54 (7.2)	
LMWH	19 (1.0)	10 (0.8)	9 (1.2)	
None	181 (9.1)	122 (9.9)	59 (7.9)	
Combined anticoagulation/ antithrombotic therapy, *n* (%)				
Triple therapy *(ASA + clopidogrel/ticagrelor + VKA/NOAC/ LMWH)*	47 (2.4)	21 (1.7)	26 (3.5)	**0**.**012**
Dual therapy *(ASA/clopidogrel/ticagrelor + VKA/NOAC/ LMWH)*	177 (8.9)	105 (8.5)	72 (9.6)	0.399
Dual antiplatelets *(ASA + clopidogrel/ticagrelor)*	8 (0.4)	3 (0.2)	5 (0.7)	0.148
Only ASA	57 (2.9)	35 (2.8)	22 (2.9)	0.893
Antiarrhythmic drugs, *n* (%)				
Sotalol	132 (6.7)	89 (7.2)	43 (5.8)	0.205
Flecainide	344 (17.4)	224 (18.2)	120 (16.1)	0.228
Amiodarone	440 (22.2)	243 (19.7)	197 (26.4)	**0**.**001**
Propafenone	8 (0.4)	6 (0.5)	2 (0.3)	0.718
Cibenzoline	1 (0.1)	1 (0.1)	0 (0.0)	1.000
None	1,067 (53.9)	679 (55.1)	388 (51.9)	0.170
Other drugs of interest, *n* (%)				
Beta-Blockers	1,421 (71.8)	877 (71.2)	544 (72.8)	0.432
Digoxin	65 (3.3)	38 (3.1)	27 (3.6)	0.521
Non-DHP calcium-channel blockers	61 (3.1)	44 (3.6)	17 (2.3)	0.106
ACE inhibitors	653 (33.0)	394 (32.0)	259 (34.7)	0.217
ARBs	361 (18.2)	233 (18.9)	128 (17.1)	0.321
Sacubitril/valsartan	47 (2.4)	25 (2.0)	22 (2.9)	0.195
Thiazide diuretics	329 (16.6)	217 (17.6)	112 (15.0)	0.129
Loop diuretics	500 (25.3)	244 (19.8)	256 (34.3)	**<0**.**001**
Aldosterone blockers	457 (23.1)	244 (19.8)	213 (28.5)	**<0**.**001**
Nitrates	80 (4.0)	39 (3.2)	41 (5.5)	**0**.**011**
DHP calcium-channel blockers	389 (19.7)	224 (18.2)	165 (22.1)	**0**.**034**
Central antihypertensive drugs	43 (2.2)	20 (1.6)	23 (3.1)	**0**.**031**
Proton pomp inhibitors	696 (35.2)	414 (33.6)	282 (37.8)	0.061
Oral antidiabetics	294 (14.9)	158 (12.8)	136 (18.2)	**0**.**001**
Insulin	87 (4.4)	41 (3.3)	46 (6.2)	**0**.**003**
Beta agonist	155 (7.8)	77 (6.3)	78 (10.4)	**0**.**001**
Anticholinergic drugs	101 (5.1)	49 (4.0)	52 (7.0)	**0**.**003**
Statins	1,108 (56.0)	689 (55.9)	419 (56.1)	0.943
Hypolipidemic non-statin drugs	161 (8.1)	102 (8.3)	59 (7.9)	0.764
Thyroid drugs	206 (10.4)	124 (10.1)	82 (11.0)	0.519
NSAIDs	22 (1.1)	17 (1.4)	5 (0.7)	0.144
Previous AF Interventions, *n* (%)				
Documented Pharmacological cardioversion	327 (16.6)	234 (19.2)	93 (12.5)	**<0**.**001**
		*(n = 1,221)*	*(n = 743)*	
Electrical cardioversion	1,139 (57.8)	724 (58.9)	415 (56.0)	0.214
		*(n = 1,230)*	*(n = 741)*	
Catheter ablation	666 (33.7)	425 (34.6)	241 (32.4)	0.325
		*(n = 1,230)*	*(n = 744)*	
Surgical therapy	15 (0.8)	8 (0.6)	7 (0.9)	0.474
LAA closure device	37 (1.9)	19 (1.5)	18 (2.4)	0.167

AF, Atrial Fibrillation; SD, Standard Deviation; BMI, Body Mass Index; CHA_2_DS_2_-VASc, Congestive heart failure(1), Hypertension (1), Age ≥75 years (2), Diabetes mellitus (1), Stroke (2), Vascular disease (1), Age 65–74 years (1), Sex category (female = 1); HAS-BLED, Systolic blood pressure >160 mmHg (1), Abnormal renal and/or hepatic function (1 point each), Stroke (1), Bleeding history or predisposition (1), Labile INR (1), Age >65 years (1), Drugs or excessive alcohol drinking (1 point each); m, male; f, female; mEHRA, modified European Heart Rhythm Association classification; NYHA, New York Heart Association functional classification; HFpEF, Heart Failure with preserved Ejection Fraction; HFmrEF, Heart Failure with midrange Ejection Fraction; HFrEF, Heart Failure with reduced Ejection Fraction; COPD, Chronic Obstructive Pulmonary Disease; OSA, Obstructive Sleep Apnea; TIA, Transient Ischemic Attack; PM, Pacemaker; ICD, Implantable Cardioverter-Defibrillator; CRT, Cardiac Resynchronization Therapy; NOAC, Non-vitamin K antagonist Oral Anticoagulant; VKA, Vitamin K Antagonist; LMWH, Low-Molecular-Weight Heparins; ASA, Acetylsalicylic Acid; NSAIDs, Nonsteroidal Anti-Inflammatory Drugs; DHP, Dihydropyridine; ACE, Angiotensin Converting Enzyme; ARB, Angiotensin Receptor Blockers; LAA, Left Atrial Appendage.

Bold indicates significant *P*-values < 0.05.

^a^ Dialysis, transplant, creatinine >2.26 mg/dL. Numbers in italics represent the number of patients in which a specific parameter was documented in their medical file.

**Figure 2:**
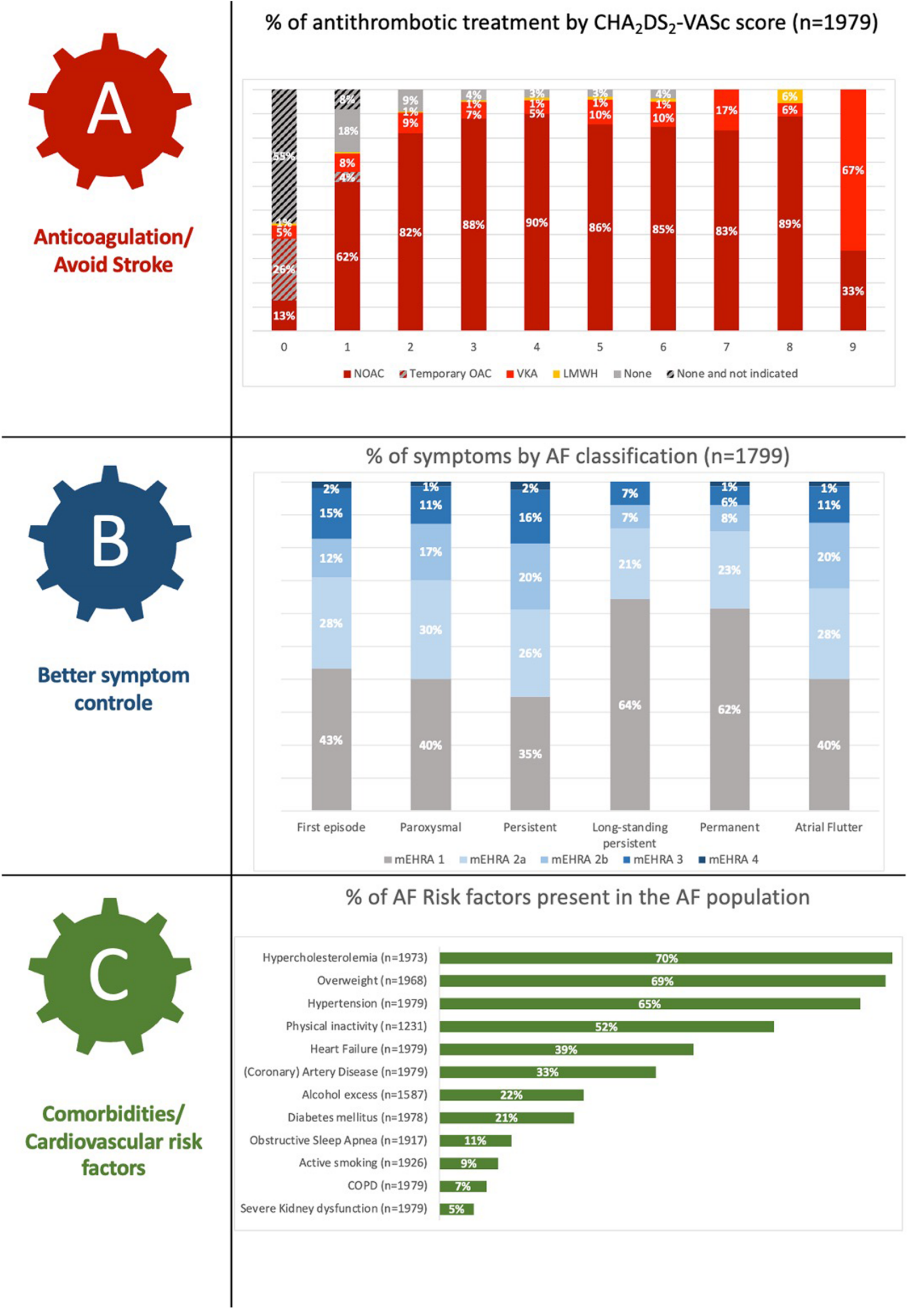
Description of the Flemish AF cohort according to the ABC pathway.

Figure 2 depicts the global characterisation of the Flemish AF population according to the “ABC pathway” focusing on anticoagulant treatment by CHA_2_DS_2_-VASc score (panel A), symptom severity by AF classification (panel B) and cardiovascular risk factors (panel C) (9). The mean CHA_2_DS_2_-VASc score was 3.4 ± 1.8 and anticoagulation therapy was prescribed in 90.9% of AF patients with the majority receiving NOACs (90.1%). A total of 112 (6.0%) out of 1,855 AF patients in whom thromboembolic prophylaxis was indicated did not receive any kind of anticoagulation therapy.

**Figure 3:**
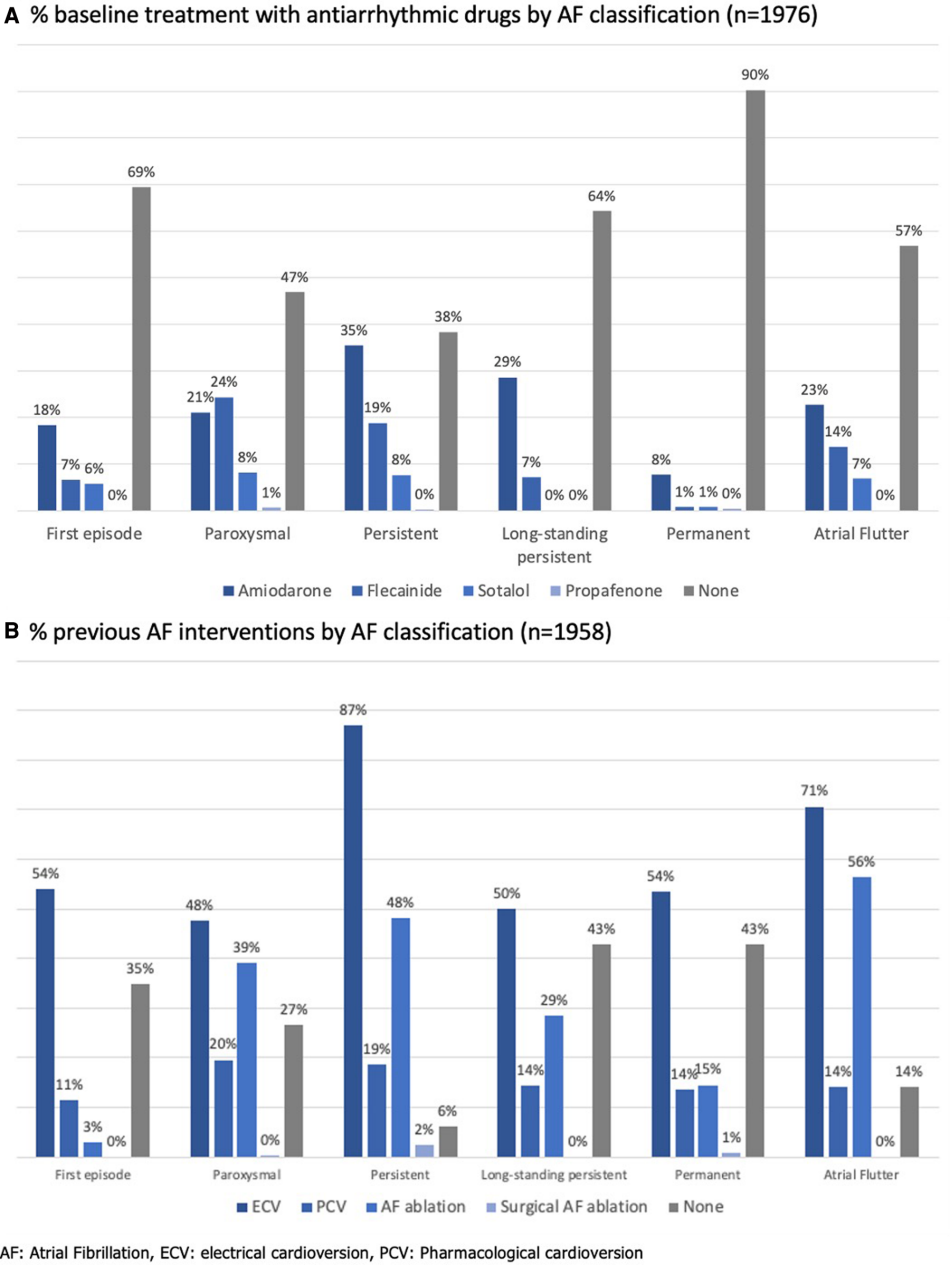
Antiarrhythmic therapy in the Flemish AF cohort. (**A**) % baseline treatment with antiarrhythmic drugs by AF classification (*n* = 1,976). (**B**) % previous AF interventions by AF classification (*n* = 1,958).

At the time of assessment for study inclusion, 42.4% of patients had no AF symptoms [scored as “1” by the modified European Heart Rhythm Association (mEHRA) symptom scale] (10). Antiarrhythmic drugs were used in 46.1% of patients and rate control was primarily obtained using beta-blockers (71.8%). Figure 3 shows the percentage of antiarrhythmics (panel A) and previous AF interventions by AF classification (panel B).

Regarding AF risk factors, overweight was present in 68.9% of the patients, 9.1% still actively smoked, 22.3% (excessively) consumed alcohol (≥8 units/week) and in only 11.2% OSA was diagnosed. Furthermore, 65.0% and 39.3% of patients were known with hypertension or a history of congestive heart failure (CHF), respectively.

### 4.3. Comparison of the study participants vs. non-participants

As shown in Table 1, there were several significant differences between the included patients and the excluded/declined patients. The majority of the non-participants were assessed while on the cardiology ward (68.1%). These patients were older (73.3 ± 9.2 years), more frequently women (41.0%), and had more comorbidities such as coronary artery disease (CAD; 37.1%), CHF (45.2%), diabetes mellitus (25.6%) and hypertension (70.7%). Consequently, these factors led to a significantly higher mean CHA_2_DS_2_-VASc score (3.8 ± 1.8) and more frequent use of diuretics, diabetic therapy and antihypertensive drugs.

### 4.4. Demographics of the AF-EduCare and AF-EduApp population

The three AF-EduCare study groups were very well balanced across the vast majority of parameters (Table 2). The AF-EduCare intervention groups were more physically active at baseline than the standard care group (*p* = 0.027). Furthermore, angiotensin-converting enzyme inhibitors (ACE-I) and thiazide diuretics were taken more often in the online education group (38.9% and 21.9%, respectively) compared with the in-person education (29.0% and 14.5%, respectively) and standard care groups (28.3% and 15.6% respectively) with *p*-values 0.004 and 0.021, respectively. Also, less use of thyroid drugs was seen in the online group (6.6%) compared to the in-person and standard care groups (both 11.6%, *p* = 0.042). Supplementary Table S1 shows the demographic characteristics of the on-treatment vs. not-eligible online education subgroups. On-treatment AF patients were higher educated, younger with fewer comorbidities such as CAD, CHF, and hypertension, resulting in lower CHA_2_DS_2_-VASc and HAS-BLED scores. This is also reflected on the therapy usage in this group with lesser use of OAC therapy (84.4% vs. 98.8%, *p* < 0.001) and diuretics and a higher prescription rate of flecainide and prior catheter ablations.

**Table 2:** Baseline characteristics of the AF-EduCare study patients.

	Total (*n* = 1,038)	In Person (*n* = 345)	Online (Intention-to-treat) (*n* = 347)	Standard Care (*n* = 346)	*P*-value
Enrolment at *outpatient* clinic, *n* (%)	493 (47.5)	167 (48.4)	163 (47.0)	163 (47.1)	0.917
AF-related, *n* (%)	436 (88.4)	147 (88.0)	152 (93.3)	137 (84.0)	**0**.**034**
Unplanned, *n* (%)	8 (1.6)	3 (1.8)	4 (2.5)	1 (0.6)	0.411
Enrolment at cardiology ward, *n* (%)	545 (52.5)	178 (51.6)	184 (53.0)	183 (52.9)	0.917
AF-related, *n* (%)	485 (89.0)	156 (87.6)	164 (89.1)	165 (90.2)	0.744
Electrical/pharmacological cardioversion	236 (48.7)	73 (46.8)	90 (54.9)	73 (44.2)	0.132
Catheter ablation	166 (34.2)	55 (35.3)	47 (28.7)	64 (38.8)	0.145
Unplanned, *n* (%)	103 (18.9)	38 (21.3)	31 (16.8)	34 (18.6)	0.545
Highest level of education, *n* (%)					0.989
Primary/Secondary school	613 (59.0)	203 (58.8)	206 (59.4)	204 (59.0)	
College/University	425 (40.9)	142 (41.2)	141 (40.6)	142 (41.0)	
Living alone, *n* (%)	220 (21.2)	75 (21.7)	70 (20.2)	75 (21.7)	0.850
Internet accessibility, *n* (%)	897 (86.4)	297 (86.1)	296 (85.3)	304 (87.9)	0.602
Independent use, *n* (%)	804 (89.6)	266 (89.6)	263 (88.9)	275 (90.5)	0.810
In possession of:					
PC/Laptop	817 (78.7)	265 (76.8)	268 (77.2)	284 (82.1)	0.170
Tablet	459 (44.2)	155 (44.9)	152 (43.8)	152 (43.9)	0.948
Smartphone	573 (55.2)	183 (53.0)	196 (56.5)	194 (56.1)	0.611
Treated by electrophysiologist, *n* (%)	611 (58.9)	213 (61.7)	201 (57.9)	197 (56.9)	0.400
Age (years), mean ± SD	69.8 ± 9.2	69.5 ± 9.3	69.9 ± 9.2	69.9 ± 9.1	0.814
Male, *n* (%)	719 (69.3)	249 (72.2)	230 (66.3)	240 (69.4)	0.244
Belgian nationality, *n* (%)	1,010 (97.3)	339 (98.3)	336 (96.8)	335 (96.8)	0.405
Race, *n* (%)					0.603
Caucasian, *n* (%)	1,032 (99.4)	342 (99.1)	345 (99.4)	345 (99.7)	
Other, *n* (%)	6 (0.6)	3 (0.9)	2 (0.6)	1 (0.3)	
BMI (kg/m^2^), mean ± SD	27.8 ± 4.9	27.7 ± 4.7	28.0 ± 4.9	27.9 ± 5.0	0.664
BMI categories, *n* (%)					0.837
≤25 kg/m^2^	319 (30.7)	104 (30.1)	107 (30.8)	108 (31.2)	
25–30 kg/m^2^	420 (40.5)	145 (42.0)	133 (38.3)	142 (41.0)	
≥30 kg/m^2^	299 (28.8)	96 (27.8)	107 (30.8)	96 (27.7)	
Kind of AF, *n* (%)					0.841
First diagnosed	141 (13.6)	42 (12.2)	49 (14.1)	50 (14.5)	
Paroxysmal AF	486 (46.8)	155 (44.9)	166 (47.8)	165 (47.7)	** **
Persistent AF	243 (23.4)	82 (23.8)	83 (23.9)	78 (22.5)	
Long-standing persistent AF	8 (0.8)	4 (1.2)	3 (0.9)	1 (0.3)	
Permanent AF	120 (11.6)	46 (13.3)	35 (10.1)	39 (11.3)	
Atrial flutter	40 (3.9)	16 (4.6)	11 (3.2)	13 (3.8)	
Time since AF diagnosis (years), mean ± SD	6.0 ± 7.3	5.9 ± 6.9	5.9 ± 7.5	6.2 ± 7.5	0.840
Rhythm at baseline, *n* (%)					0.284
Sinus rhythm	793 (76.4)	257 (74.5)	261 (75.2)	275 (79.5)	
AF/Atrial flutter	222 (21.4)	79 (22.9)	81 (23.4)	62 (17.9)	
Other rhythm	23 (2.2)	9 (2.6)	5 (1.4)	9 (2.6)	
CHA_2_DS_2_-VASc score, mean ± SD	3.1 ± 1.7	3.1 ± 1.7	3.3 ± 1.7	3.1 ± 1.7	0.288
CHA_2_DS_2_-VASc classification, *n* (%)					0.943
CHA_2_DS_2_-VASc score 0 (m) or 1 (f)	77 (7.4)	28 (8.1)	23 (6.6)	26 (7.5)	
CHA_2_DS_2_-VASc score 1 (m) or 2 (f)	160 (15.4)	55 (15.9)	52 (15.0)	53 (15.3)	
CHA_2_DS_2_-VASc score ≥ 2 (m) or ≥3 (f)	801 (77.2)	262 (75.9)	272 (78.4)	267 (77.2)	
HAS-BLED score, mean ± SD	1.5 ± 0.9	1.5 ± 0.9	1.5 ± 0.9	1.5 ± 0.9	0.598
HAS-BLED classification, *n* (%)					0.905
HAS-BLED 0–2	909 (87.6)	304 (88.1)	304 (87.6)	301 (87.0)	
HAS-BLED ≥ 3	129 (12.4)	41 (11.9)	43 (12.4)	45 (13.0)	
mEHRA, *n* (%)			** **	** **	0.999
1	430 (41.4)	141 (40.9)	142 (40.9)	147 (42.5)	** **
2a	300 (28.9)	99 (28.7)	104 (30.0)	97 (28.0)	** **
2b	177 (17.1)	59 (17.1)	59 (17.0)	59 (17.1)	
3	118 (11.4)	42 (12.2)	37 (10.7)	39 (11.3)	
4	13 (1.3)	4 (1.2)	5 (1.4)	4 (1.2)	
Concomitant disease, *n* (%)					
(Coronary) artery disease	323 (31.1)	108 (31.3)	111 (32.0)	104 (30.1)	0.857
History of congestive heart failure	376 (36.2)	125 (36.2)	135 (38.9)	116 (33.5)	0.338
NYHA class III/IV	75 (20.0)	26 (20.8)	27 (20.0)	22 (18.9)	0.949
Heart failure classification					0.874
HFpEF	186 (49.6)	59 (47.2)	69 (51.1)	58 (50.0)	
HFmrEF	80 (21.3)	30 (24.0)	25 (18.5)	25 (21.6)	
HFrEF	110 (29.1)	36 (28.8)	41 (30.4)	33 (28.4)	
Thyroid disease					0.052
Hyperthyroidism	52 (5.0)	23 (6.7)	9 (2.6)	20 (5.8)	
Hypothyroidism	97 (9.3)	36 (10.4)	26 (7.5)	35 (10.1)	
Severe kidney dysfunction^a^	38 (3.7)	14 (4.1)	11 (3.2)	13 (3.8)	0.819
COPD	65 (6.3)	18 (5.2)	20 (5.8)	27 (7.8)	0.335
Active malignancy	30 (2.9)	8 (2.3)	15 (4.3)	7 (2.0)	0.145
Liver disease	7 (0.7)	3 (0.9)	1 (0.3)	3 (0.9)	0.581
Cardiovascular risk factors, *n* (%)					
Diabetes mellitus type I/II	184 (17.7)	59 (17.1)	60 (17.3)	65 (18.8)	0.817
Hypertension	634 (61.1)	206 (59.7)	226 (65.1)	202 (58.4)	0.155
Hypercholesterolemia^b^	713 (68.9)	234 (68.4)	233 (67.1)	246 (71.1)	0.518
Current smoker	79 (7.6)	23 (6.7)	33 (9.5)	23 (6.6)	0.474
Alcohol excess (≥8/week)	240 (23.1)	90 (26.1)	66 (19.0)	84 (24.3)	0.072
Documented diagnosis of OSA^c^	125 (12.3)	37 (11.0)	39 (11.5)	49 (14.3)	0.382
Physical inactivity (<60 min/week)^d^	551 (53.1)	176 (51.0)	171 (49.4)	204 (59.0)	**0**.**027**
Co-morbidities, *n* (%)					
Previous TIA	63 (6.1)	20 (5.8)	25 (7.2)	18 (5.2)	0.526
Previous ischaemic stroke	71 (6.8)	23 (6.7)	21 (6.1)	27 (7.8)	0.651
Previous hemorrhagic stroke	2 (0.2)	0 (0.0)	1 (0.3)	1 (0.3)	1.000
Other Ischaemic thrombo-embolic events	9 (0.9)	3 (0.9)	3 (0.9)	3 (0.9)	1.000
History of pulmonary embolism	18 (1.7)	6 (1.7)	5 (1.4)	7 (2.0)	0.842
Bleeding history	18 (1.7)	2 (0.6)	9 (2.6)	7 (2.0)	0.099
Devices					0.844
PM	82 (7.9)	31 (9.0)	25 (7.2)	26 (7.5)	
ICD	52 (5.0)	20 (5.8)	18 (5.2)	14 (4.0)	
CRT-PM	16 (1.5)	4 (1.2)	5 (1.4)	7 (2.0)	
CRT-ICD	23 (2.2)	10 (2.9)	8 (2.3)	5 (1.4)	
Anticoagulation therapy, *n* (%)					0.485
NOAC	832 (80.2)	276 (80.0)	279 (80.4)	277 (80.1)	
Apixaban	264 (31.7)	72 (26.1)	85 (30.5)	107 (38.6)	0.011
Edoxaban	276 (33.2)	92 (33.3)	105 (37.6)	79 (28.5)	
Rivaroxaban	210 (25.2)	86 (31.2)	60 (21.5)	64 (23.1)	
Dabigatran	82 (9.9)	26 (9.4)	29 (10.4)	27 (9.7)	
VKA	89 (8.6)	33 (9.6)	25 (7.2)	31 (9.0)	
LMWH	10 (1.0)	4 (1.2)	1 (0.3)	5 (1.4)	
None	107 (10.3)	32 (9.3)	42 (12.1)	33 (9.5)	
Combined anticoagulation/ antithrombotic therapy, *n* (%)					
Triple therapy *(ASA + clopidogrel/ticagrelor + VKA/NOAC/ LMWH)*	17 (1.6)	7 (2.0)	6 (1.7)	4 (1.2)	0.656
Dual therapy *(ASA/clopidogrel/ticagrelor + VKA/NOAC/ LMWH)*	83 (8.0)	29 (8.4)	29 (8.4)	25 (7.2)	0.811
Dual antiplatelets *(ASA + clopidogrel/ticagrelor)*	3 (0.3)	2 (0.6)	0 (0.0)	1 (0.3)	0.221
Only ASA	31 (3.0)	11 (3.2)	12 (3.5)	8 (2.3)	0.651
Antiarrhythmic drugs, *n* (%)					
Sotalol	77 (7.4)	22 (6.4)	30 (8.6)	25 (7.2)	0.516
Flecainide	188 (18.1)	62 (18.0)	67 (19.3)	59 (17.1)	0.740
Amiodarone	202 (19.5)	66 (19.1)	56 (16.1)	80 (23.1)	0.066
Propafenone	5 (0.5)	3 (0.9)	1 (0.3)	1 (0.3)	0.463
Cibenzoline	1 (0.1)	0 (0.0)	0 (0.0)	1 (0.1)	0.666
None	574 (55.3)	194 (56.2)	197 (56.8)	183 (52.9)	0.538
Other drugs of interest, *n* (%)					
Beta-Blockers	736 (70.9)	249 (72.2)	246 (70.9)	241 (69.7)	0.766
Digoxin	35 (3.4)	15 (4.3)	11 (3.2)	9 (2.6)	0.431
Non-DHP calcium-channel blockers	40 (3.9)	13 (3.8)	12 (3.5)	15 (4.3)	0.831
ACE inhibitors	333 (32.1)	100 (29.0)	135 (38.9)	98 (28.3)	**0**.**004**
ARBs	187 (18.0)	55 (15.9)	72 (20.7)	60 (17.3)	0.239
Sacubitril/valsartan	20 (1.9)	10 (2.9)	7 (2.0)	3 (0.9)	0.152
Thiazide diuretics	180 (17.3)	50 (14.5)	76 (21.9)	54 (15.6)	**0**.**021**
Loop diuretics	205 (19.7)	70 (20.3)	64 (18.4)	71 (20.5)	0.753
Aldosterone blockers	200 (19.3)	69 (20.0)	69 (19.9)	62 (17.9)	0.738
Nitrates	34 (3.3)	14 (4.1)	6 (1.7)	14 (4.0)	0.140
DHP calcium-channel blockers	182 (17.5)	55 (15.9)	69 (19.9)	58 (16.8)	0.355
Central antihypertensive drugs	18 (1.7)	5 (1.4)	7 (2.0)	6 (1.7)	0.849
Proton pomp inhibitors	344 (33.1)	120 (34.8)	111 (32.0)	113 (32.7)	0.718
Oral antidiabetics	128 (12.3)	41 (11.9)	45 (13.0)	42 (12.1)	0.857
Insulin	36 (3.5)	10 (2.9)	13 (3.7)	13 (3.8)	0.778
Beta agonist	61 (5.9)	15 (4.3)	24 (6.9)	22 (6.4)	0.320
Anticholinergic drugs	42 (4.0)	9 (2.6)	17 (4.9)	16 (4.6)	0.249
Statins	583 (56.2)	191 (55.4)	202 (58.2)	190 (54.9)	0.637
Hypolipidemic non-statin drugs	83 (8.0)	24 (7.0)	27 (7.8)	32 (9.2)	0.531
Thyroid drugs	103 (9.9)	40 (11.6)	23 (6.6)	40 (11.6)	**0**.**042**
NSAIDs	13 (1.3)	3 (0.9)	5 (1.4)	5 (1.4)	0.829
AF Interventions, *n* (%)					
Documented Pharmacological cardioversion^e^	205 (19.9)	72 (21.2)	63 (18.2)	70 (20.4)	0.587
Electrical cardioversion^f^	613 (59.1)	210 (60.9)	194 (55.9)	209 (60.6)	0.329
Catheter ablation^g^	355 (34.2)	124 (36.0)	108 (31.1)	123 (35.5)	0.323
Surgical therapy	7 (0.7)	0 (0.0)	4 (1.2)	3 (0.9)	0.172
LAA closure device	14 (1.3)	3 (0.9)	4 (1.2)	7 (2.0)	0.391

AF, Atrial Fibrillation; SD, Standard Deviation; BMI, Body Mass Index; CHA_2_DS_2_-VASc, Congestive heart failure(1), Hypertension (1), Age ≥75 years (2), Diabetes mellitus (1), Stroke (2), Vascular disease (1), Age 65–74 years (1), Sex category (female = 1); HAS-BLED, Systolic blood pressure >160 mmHg (1), Abnormal renal and/or hepatic function (1 point each), Stroke (1), Bleeding history or predisposition (1), Labile INR (1), Age >65 years (1), Drugs or excessive alcohol drinking (1 point each); m, male; f, female; mEHRA, modified European Heart Rhythm Association classification; NYHA, New York Heart Association functional classification; HFpEF, Heart Failure with preserved Ejection Fraction; HFmrEF, Heart Failure with midrange Ejection Fraction; HFrEF, Heart Failure with reduced Ejection Fraction; COPD, Chronic Obstructive Pulmonary Disease; OSA, Obstructive Sleep Apnea; TIA, Transient Ischemic Attack; PM, Pacemaker; ICD, Implantable Cardioverter-Defibrillator; CRT, Cardiac Resynchronization Therapy; NOAC, Non-vitamin K antagonist Oral Anticoagulant; VKA, Vitamin K Antagonist; LMWH, Low-Molecular-Weight Heparins; ASA, Acetylsalicylic Acid; NSAIDs, Nonsteroidal Anti-Inflammatory Drugs; DHP, Dihydropyridine; ACE, Angiotensin Converting Enzyme; ARB, Angiotensin Receptor Blockers; LAA, Left Atrial Appendage.

Bold indicates significant *P*-values < 0.05.

^a^ Dialysis, transplant, creatinine > 2.26 mg/dL.

^b^ 3 unknown.

^c^ 21 unknown.

^d^ 1 unknown.

^e^ 8 unknown.

^f^ 1 unknown.

^g^ 1 unknown.

Both the AF-EduCare and AF-EduApp study populations were also comparable across the demographic parameters (Table 3). Remarkably, 23.1% and 30.4% of the AF-EduCare and AF-EduApp study patients respectively, consumed ≥8 alcoholic beverages/week (*p* = 0.030). The AF-EduApp group was physically more active than the AF-EduCare group (*p* = 0.017). Supplementary Table S2 shows the demographic characteristics of the on-treatment vs. not-eligible application subgroups. Several significant differences were seen including younger age, lower CHA_2_DS_2_-VASc score, more first diagnosed and less permanent AF patients, less diabetes mellitus, less hypercholesterolemia (with less use of statins), less treatment with antiarrhythmic drugs in the on-treatment group, in line with expectations for a group of patients that accept intervention with a mobile Health (mHealth) app.

**Table 3:** Comparison AF-EduCare vs. AF-EduApp population.

	Included (*n* = 1,232)	AF-EduCare (*n* = 1,038)	AF-EduApp Intention-to-treat (*n* = 194)	*P*-value
Enrolment at outpatient clinic, *n* (%)	569 (46.2)	493 (47.5)	76 (39.2)	**0**.**033**
AF-related, *n* (%)	505 (88.8)	436 (88.4)	69 (90.8)	0.546
Unplanned, *n* (%)	9 (1.6)	8 (1.6)	1 (1.3)	1.000
Enrolment at cardiology ward, *n* (%)	663 (53.8)	545 (52.5)	118 (60.8)	**0**.**033**
AF-related, *n* (%)	587 (88.5)	485 (89.0)	102 (86.4)	0.430
Electrical/pharmacological cardioversion	287 (48.9)	236 (48.7)	51 (50.0)	0.806
Catheter ablation	196 (33.4)	166 (34.2)	30 (29.4)	0.349
Unplanned, *n* (%)	133 (20.1)	103 (18.9)	30 (25.4)	0.109
Highest level of education, *n* (%)				0.541
Primary/Secondary school	723 (58.7)	613 (59.0)	110 (56.7)	
College/University	509 (41.3)	425 (40.9)	84 (43.3)	
Living alone, *n* (%)	260 (21.1)	220 (21.2)	40 (20.6)	0.857
Internet accessibility, *n* (%)	1,078 (87.5)	897 (86.4)	181 (93.3)	**0**.**008**
Independent use, *n* (%)	980 (90.9)	804 (89.6)	176 (97.2)	**0**.**001**
In possession of:				
PC/Laptop	971 (78.8)	817 (78.7)	154 (79.4)	0.833
Tablet	564 (45.8)	459 (44.2)	105 (54.1)	**0**.**011**
Smartphone	729 (59.2)	573 (55.2)	156 (80.4)	**<0**.**001**
Treated by electrophysiologist, *n* (%)	697 (56.6)	611 (58.9)	86 (44.3)	**<0**.**001**
Age (years), mean ± SD	69.8 ± 8.9	69.8 ± 9.2	70.1 ± 7.0	0.554
Male, *n* (%)	851 (69.1)	719 (69.3)	132 (68.0)	0.734
Belgian nationality, *n* (%)	1,201 (97.5)	1,010 (97.3)	191 (98.5)	0.347
Race, *n* (%)				1.000
Caucasian, *n* (%)	1,225 (99.4)	1,032 (99.4)	193 (99.5)	
Other, *n* (%)	7 (0.6)	6 (0.6)	1 (0.5)	
BMI (kg/m^2^), mean ± SD	27.9 ± 4.9	27.8 ± 4.9	28.5 ± 5.3	0.103
BMI categories, *n* (%)				0.180
≤25 kg/m^2^	366 (29.7)	319 (30.7)	47 (24.2)	
25–30 kg/m^2^	508 (41.2)	420 (40.5)	88 (45.4)	
≥30 kg/m^2^	358 (29.1)	299 (28.8)	59 (30.4)	
Kind of AF, *n* (%)				0.767
First diagnosed	171 (13.9)	141 (13.6)	30 (15.5)	
Paroxysmal AF	584 (47.4)	486 (46.8)	98 (50.5)	
Persistent AF	281 (22.8)	243 (23.4)	38 (19.6)	
Long-standing persistent AF	9 (0.7)	8 (0.8)	1 (0.5)	
Permanent AF	139 (11.3)	120 (11.6)	19 (9.8)	
Atrial flutter	48 (3.9)	40 (3.9)	8 (4.1)	
Time since AF diagnosis (years), mean ± SD	5.8 ± 7.2	6.0 ± 7.3	5.1 ± 6.4	0.076
Rhythm at baseline, *n* (%)				0.135
Sinus rhythm	932 (75.7)	792 (76.73	140 (72.2)	
AF/Atrial flutter	275 (22.3)	222 (21.4)	53 (27.3)	
Other rhythm	24 (1.9)	23 (2.2)	1 (0.1)	
CHA_2_DS_2_-VASc score, mean ± SD	3.1 ± 1.7	3.1 ± 1.7	3.1 ± 1.6	0.945
CHA_2_DS_2_-VASc classification, *n* (%)				0.559
CHA_2_DS_2_-VASc score 0 (m) or 1 (f)	88 (7.1)	77 (7.4)	11 (5.7)	
CHA_2_DS_2_-VASc score 1 (m) or 2 (f)	187 (15.2)	160 (15.4)	27 (13.9)	
CHA_2_DS_2_-VASc score ≥ 2 (m) or ≥3 (f)	957 (77.7)	801 (77.2)	156 (80.4)	
HAS-BLED score, mean ± SD	1.5 ± 0.9	1.5 ± 0.9	1.6 ± 0.9	**0**.**034**
HAS-BLED classification, *n* (%)				0.442
HAS-BLED 0–2	1,075 (87.3)	909 (87.6)	166 (85.6)	** **
HAS-BLED ≥ 3	157 (12.7)	129 (12.4)	28 (14.4)	** **
mEHRA, *n* (%)				0.682
1	514 (41.7)	430 (41.4)	84 (43.3)	
2a	361 (29.3)	300 (28.9)	61 (31.4)	
2b	204 (16.6)	177 (17.1)	27 (13.9)	
3	139 (11.3)	118 (11.4)	21 (10.8)	
4	14 (1.1)	13 (1.3)	1 (0.5)	
Concomitant disease, *n* (%)				
(Coronary) artery disease	385 (31.3)	323 (31.1)	62 (32.0)	0.817
History of congestive heart failure	439 (35.6)	376 (36.2)	63 (32.5)	0.317
NYHA class III/IV	91 (20.9)	75 (20.0)	16 (25.8)	0.297
Heart failure classification				0.488
HFpEF	213 (48.5)	186 (49.5)	27 (42.9)	
HFmrEF	93 (21.2)	80 (21.3)	13 (20.6)	
HFrEF	133 (30.3)	110 (29.3)	23 (36.5)	
Thyroid disease				0.305
Hyperthyroidism	67 (5.4)	52 (5.0)	15 (7.7)	
Hypothyroidism	114 (9.3)	97 (9.3)	17 (8.8)	
Severe kidney dysfunction^a^	46 (3.7)	38 (3.7)	8 (4.1)	0.755
COPD	81 (6.6)	65 (6.3)	16 (8.2)	0.306
Active malignancy	35 (2.8)	30 (2.9)	5 (2.6)	0.810
Liver disease	9 (0.7)	7 (0.7)	2 (1.0)	0.639
Cardiovascular risk factors, *n* (%)				
Diabetes mellitus type I/II	220 (17.9)	184 (17.7)	36 (18.6)	0.782
Hypertension	759 (61.6)	634 (61.1)	125 (64.4)	0.378
Hypercholesterolemia^b^	848 (69.1)	713 (68.9)	135 (69.9)	0.770
Current smoker	99 (8.0)	79 (7.6)	20 (10.3)	0.204
Alcohol excess (≥8/week)	299 (24.3)	240 (23.1)	59 (30.4)	**0**.**030**
Documented diagnosis of OSA^c^	152 (12.6)	125 (12.3)	27 (14.1)	0.497
Physical inactivity (<60 min/week)^d^	636 (51.7)	551 (53.1)	85 (43.8)	**0**.**017**
Co-morbidities, *n* (%)				
Previous TIA	78 (6.3)	63 (6.1)	15 (7.7)	0.383
Previous ischaemic stroke	83 (6.7)	71 (6.8)	12 (6.2)	0.739
Previous hemorrhagic stroke	3 (0.2)	2 (0.2)	1 (0.5)	0.402
Other Ischaemic thrombo-embolic events	9 (0.7)	9 (0.9)	0 (0.0)	0.369
History of pulmonary embolism	19 (1.5)	18 (1.7)	1 (0.5)	0.340
Bleeding history	26 (2.1)	18 (1.7)	8 (4.1)	**0**.**034**
Devices				0.697
PM	99 (8.0)	82 (7.9)	17 (8.8)	
ICD	58 (4.7)	52 (5.0)	6 (3.1)	
CRT-PM	18 (1.5)	16 (1.5)	2 (1.0)	
CRT-ICD	25 (2.0)	23 (2.2)	2 (1.0)	
Anticoagulation therapy, *n* (%)				0.349
NOAC	995 (80.8)	832 (80.2)	163 (84.0)	
Apixaban	316 (31.8)	264 (31.7)	52 (31.9)	0.258
Edoxaban	339 (34.1)	276 (33.2)	63 (38.7)	
Rivaroxaban	240 (24.1)	210 (25.2)	30 (18.4)	
Dabigatran	100 (10.1)	82 (9.9)	18 (11.0)	
VKA	105 (8.5)	89 (8.6)	16 (8.2)	
LMWH	10 (0.8)	10 (1.0)	0 (0.0)	
None	122 (9.9)	107 (10.3)	15 (7.7)	
Combined anticoagulation/ antithrombotic therapy, *n* (%)				
Triple therapy *(ASA + clopidogrel/ticagrelor + VKA/NOAC/ LMWH)*	21 (1.7)	17 (1.6)	4 (2.1)	0.761
Dual therapy *(ASA/clopidogrel/ticagrelor + VKA/NOAC/ LMWH)*	105 (8.5)	83 (8.0)	22 (11.3)	0.126
Dual antiplatelets *(ASA + clopidogrel/ticagrelor)*	3 (0.2)	3 (0.3)	0 (0.0)	1.000
Only ASA	35 (2.8)	31 (3.0)	4 (2.1)	0.639
Antiarrhythmic drugs, *n* (%)				
Sotalol	89 (7.2)	77 (7.4)	12 (6.2)	0.543
Flecainide	224 (18.2)	188 (18.1)	36 (18.6)	0.883
Amiodarone	243 (19.7)	202 (19.5)	41 (21.1)	0.591
Propafenone	6 (0.5)	5 (0.5)	1 (0.5)	1.000
Cibenzoline	1 (0.1)	1 (0.1)	0 (0.0)	1.000
None	679 (55.1)	574 (55.3)	105 (54.1)	0.763
Other drugs of interest, *n* (%)				
Beta-Blockers	877 (71.2)	736 (70.9)	141 (72.7)	0.616
Digoxin	38 (3.1)	35 (3.4)	3 (1.5)	0.256
Non-DHP calcium-channel blockers	44 (3.6)	40 (3.9)	4 (2.1)	0.292
ACE inhibitors	394 (32.0)	333 (32.1)	61 (31.4)	0.861
ARBs	233 (18.9)	187 (18.0)	46 (23.7)	0.063
Sacubitril/valsartan	25 (2.0)	20 (1.9)	5 (2.6)	0.555
Thiazide diuretics	217 (17.6)	180 (17.3)	37 (19.1)	0.561
Loop diuretics	244 (19.8)	205 (19.7)	39 (20.1)	0.910
Aldosterone blockers	244 (19.8)	200 (19.3)	44 (22.7)	0.274
Nitrates	39 (3.2)	34 (3.3)	5 (2.6)	0.610
DHP calcium-channel blockers	224 (18.2)	182 (17.5)	42 (21.6)	0.172
Central antihypertensive drugs	20 (1.6)	18 (1.7)	2 (1.0)	0.756
Proton pomp inhibitors	414 (33.6)	344 (33.1)	70 (36.1)	0.426
Oral antidiabetics	158 (12.8)	128 (12.3)	30 (15.5)	0.231
Insulin	41 (3.3)	36 (3.5)	5 (2.6)	0.525
Beta agonist	77 (6.3)	61 (5.9)	16 (8.2)	0.211
Anticholinergic drugs	49 (4.0)	42 (4.0)	7 (3.6)	0.774
Statins	689 (55.9)	583 (56.2)	106 (54.6)	0.694
Hypolipidemic non-statin drugs	102 (8.3)	83 (8.0)	19 (9.8)	0.404
Thyroid drugs	124 (10.1)	103 (9.9)	21 (10.8)	0.702
NSAIDs	17 (1.4)	13 (1.3)	4 (2.1)	0.326
AF Interventions, *n* (%)				
Documented Pharmacological cardioversion^e^	234 (19.2)	205 (19.9)	29 (15.2)	0.128
Electrical cardioversion^f^	724 (58.9)	613 (59.1)	111 (57.5)	0.678
Catheter ablation^g^	425 (34.6)	355 (34.2)	70 (36.3)	0.585
Surgical therapy	8 (0.6)	7 (0.74	1 (0.5)	1.000
LAA closure device	19 (1.5)	14 (1.4)	5 (2.6)	0.199

AF, Atrial Fibrillation, SD, Standard Deviation, BMI, Body Mass Index, CHA_2_DS_2_-VASc, Congestive heart failure(1), Hypertension (1), Age ≥75 years (2), Diabetes mellitus (1), Stroke (2), Vascular disease (1), Age 65–74 years (1), Sex category (female = 1); HAS-BLED: Systolic blood pressure >160 mmHg (1), Abnormal renal and/or hepatic function (1 point each), Stroke (1), Bleeding history or predisposition (1), Labile INR (1), Age >65 years (1), Drugs or excessive alcohol drinking (1 point each), m, male, f, female, mEHRA, modified European Heart Rhythm Association classification, NYHA, New York Heart Association functional classification, HFpEF, Heart Failure with preserved Ejection Fraction, HFmrEF, Heart Failure with midrange Ejection Fraction, HFrEF, Heart Failure with reduced Ejection Fraction, COPD, Chronic Obstructive Pulmonary Disease, OSA, Obstructive Sleep Apnea, TIA, Transient Ischemic Attack, PM, Pacemaker, ICD, Implantable Cardioverter-Defibrillator, CRT, Cardiac Resynchronization Therapy, NOAC, Non-vitamin K antagonist Oral Anticoagulant, VKA, Vitamin K Antagonist, LMWH, Low-Molecular-Weight Heparins, ASA, Acetylsalicylic Acid, NSAIDs, Nonsteroidal Anti-Inflammatory Drugs, DHP, Dihydropyridine, ACE, Angiotensin Converting Enzyme, ARB, Angiotensin Receptor Blockers, LAA, Left Atrial Appendage.

Bold indicates significant *P*-values < 0.05.

^a^ Dialysis, transplant, creatinine > 2.26 mg/dL.

^b^ 4 unknown.

^c^ 23 unknown.

^d^ 1 unknown.

^e^ 11 unknown.

^f^ 2 unknown.

^g^ 2 unknown.

Regarding internet accessibility and multimedia, a high proportion of the included patients had internet access (87.5%) and 90.9% of these patients could independently work with it. When comparing the AF-EduCare group with the AF-EduApp group, study patients of the latter group had significantly more internet accessibility and more possession of a tablet (54.1% vs. 44.2%, *p* = 0.011) or a smartphone (80.4% vs. 55.2%, *p* < 0.001).

## 5. Discussion

Our results provide data from the largest and most recent contemporary AF population in Flanders (the Dutch-speaking north of Belgium) regarding comorbidities and current management. As AF prevalence is anticipated to rise further in the coming decades, it is important to have up-to-date insights into AF population characteristics and current AF management. Compared with international registries, there is a higher use of anticoagulation (mainly with NOACs), conform the most recent guidelines. Also rhythm control therapies were more frequently applied, both pharmacologically and especially ablation.

The education and integrated care intervention of the AF-EduCare/AF-EduApp study was targeted at an unselected AF population, in contrast to previous integrated care trials which included selected AF patient populations (11–18). Nevertheless, almost 35% of the patients had to be excluded or declined participation, illustrating the difficulties in reaching all AF patients with integrated care approaches. The clinical outcome events in this large AF study cohort will provide extra information on the effectiveness and best strategies to implement integrated AF care.

### 5.1. Current flemish AF population compared to other cohorts

When comparing our cohort with other European/Western prospective studies and international registries, similarities and important evolutions can be noticed (Supplementary Table S3).

Concerning the general demographic characteristics, our total cohorts' mean age of 71.2 years was similar to other registries ranging between 68.8–73.5 years old (19–25). Our population showed a slightly higher proportion of men (65.3%) compared with other studies (range between 55%–60%) (19–25). In our study, 59.2% of AF patients were approached at the cardiology ward, whereas the EURObservational Research Programme on Atrial Fibrillation (EORP-AF) Pilot and Long-Term General Registries included 62.8% and 52.2% hospitalised AF patients, respectively (19, 20).

The management of AF shows several evolutions. In line with the “ABC” pathway as proposed in the 2020 ESC guidelines, an important aspect in the treatment of AF is stroke prevention (**“A—Anticoagulation/Avoid stroke”**) in patients with high thromboembolic risk, for which NOACs are now the preferred therapy over vitamin K antagonists (VKA) (4). Therapy with NOACs and VKAs were prescribed in 81.9% and 8.0% of our cohort, respectively. AF patients with increasing thromboembolic risk (i.e., CHA_2_DS_2_-VASc score) were more increasingly treated with OAC, which reflects a good adherence to the Guidelines (Figure 2) (4). The use of OACs in AF patients with CHA_2_DS_2_-VASc score 0 (male) or 1 (female) could partly be explained by the enrolment of patients with a mechanical heart valve or who recently underwent direct current cardioversion or AF ablation for which temporary OAC use was indicated (26). Of the 255 patients with a CHA_2_DS_2_-VASc score of 1 (male) or 2 (female), 45 (17.6%) were not receiving OAC, although this therapy should be considered in these patients (Class IIa, level B recommendation) (4). The AF stroke risk factors that were most prevalent In this patient group were age (65–75 years old; 45.5%) and hypertension (35.3%), which are both clearly recognised as risk markers which on their own justify anticoagulation. Prior (Western-)European registries reported OAC use in 73.0%–85.0% of AF patients, and lower use of NOACs, although there is a clear temporal trend for increase in the more recent studies (19–24).

For **“B—Better symptom control”**, beta-blockers were primarily used as rate control therapy conform other registries. Digoxin was only prescribed in 3.3% of our cohort compared to 14.7% in the latest EORP-AF long-term registry (20).

Remarkably, in our cohort, 53% and 62% of the paroxysmal AF and persistent AF patients respectively took antiarrhythmic drugs. The majority of both patient groups had also undergone a previous AF intervention. Only 40% and 35% respectively were asymptomatic at the time of inclusion in our cohorts. In the EORP-AF long-term general registry, less antiarrhythmics were prescribed for paroxysmal (44.0%) and persistent AF (42.5%) patients, although the asymptomatic proportion in these patients were similar to our cohort (42.7% and 35.2%, respectively) (20). Overall, rhythm control was pursued more in this Flemish cohort than in the EORP-AF population, since cardioversions and ablations were performed in 65.5% vs. 42.2%, and 34.5% vs. 5.8% of patients. Moreover, 40.3% of these patients were enrolled during an admission for AF ablation or cardioversion.

The third pillar of the ABC pathway, namely “**C—Comorbidities/Cardiovascular risk factors**”, showed interesting findings in the Flemish population. Being overweight forms a major problem (mean BMI 28.0 kg/m^2^), comparable with international registries (BMI range 27.7–31.2 kg/m^2^) (19, 23, 24). We noted a slightly lower prevalence of hypertension (65.0% vs. >70% in the majority of registries) (19–25). Our findings are in line with the hypertension prevalence noted in the regional subanalysis of the ETNA-AF Europe study (61.6%), GARFIELD-AF (68.3%), and EORP-AF long-term general registry (49.8%). It indicates that other AF risk factors play a more important role in Belgian patients. Our study recorded a higher presence of hypercholesterolemia in our AF patients (70.3%) than the two EORP-AF registries (41%–48%) (19, 20). Furthermore, when systematically asking about AF patients' lifestyle, a rather high prevalence of regular alcohol consumption (≥8 units/week) was noted in almost a quarter of our included AF patients. This risk factor may not be underestimated as moderate to heavy alcohol consumption is related to AF progression and recurrence (27, 28). Lastly, the likely underrecognition of OSA (11.2%) is noteworthy, as the prevalence of even moderate OSA in AF patients has been estimated to be between 42.1%–56.1% (29). This calls for better screening of OSA in Flemish (and other) AF patient populations.

### 5.2. Patients' willingness to participate in integrated AF care programs

Thirty-three percent of the eligible AF patients could not, or refused to, participate in the AF-EduCare/AF-EduApp study. This high rate can partly be explained by the design of the trial: candidates could not choose their preferred treatment group, and the possibility for extra in-hospital study visits (i.e., in-person education group) was perceived as difficult, leading to patient refusal. This is reflected in the primary reason for non-participation, i.e., transportation problems. Hence, future integrated AF programs should offer at least the possibility for remote care through teleconsultations and mHealth technology (e.g., telemonitoring of blood pressure, heart rate and rhythm,…). This technology opportunity has also been the reason to implement the additional AF-EduApp study arm to gain experience and to evaluate intermediate patient-related outcomes when using mHealth technology. The COVID-19 pandemic certainly has accelerated the development, validation and use of remote AF technology which could support integrated AF care (30). Many challenges still await concerning remote health technology, as extensively discussed in the international collaborative statement paper by Varma et al. (31).

On the other hand, when looking at the clinical profile of the non-participating patients, who were older, were more often hospitalised (i.e., sicker), and often had limited access to or affinity with mHealth, such AF patients may derive most benefit from integrated care. This group is more vulnerable to complications due to its higher prevalence of comorbidities and AF risk factors. These patients, therefore, should not be forgotten and care pathways that motivate them for integrated AF care should be explored.

### 5.3. Comparison of the AF-EduCare/AF-EduApp cohort with other integrated AF care trials

Over the last decade, several integrated AF care trials were conducted with varying success on clinical outcomes (Supplementary Table S4) (11–18). These studies show important differences in the characteristics of the included AF patients.

The RCT by Hendriks et al., the study by Carter et al. and the RACE 4 trial included AF patients with a new AF diagnosis and/or patients seen at the outpatient clinic (11, 13, 14). Consequently, these patients were younger and had fewer comorbidities, resulting in a lower overall thromboembolic risk (range CHA_2_DS_2_-VASc score 2.2–2.4). OACs were taken in 57.0 to 67.6% of patients.

In contrast to these three studies, the SAFETY trial included hospitalised patients with chronic AF but without chronic heart failure (12). This cohort had a mean age of 72 years and had more comorbidities. Only VKAs were available at that time and were prescribed in just 55.5% of patients despite a mean CHA_2_DS_2_-VASc score of 3.6.

Two recent integrated AF trials conducted in primary care included older patients but with variable prevalence of comorbidities compared with our results, i.e., CHF, diabetes mellitus and TIA/stroke in 16.8%−25.7%, 25.5%–28.9% and 14.4%–18.4% of patients, respectively (15, 16). OAC was used in the 71.3%–90.7% of patients.

Lastly, the mAFA-II trial included in- and outpatient candidates but with a CHA_2_DS_2_-VASc score ≥2 (18). This population was slightly younger (mean 68.5 years old) compared to the AF-EduCare/AF-EduApp population. Comorbidities were generally less common except for CAD which was more prevalent in the mAFA population (40.9%) compared with our included study population. Median CHA_2_DS_2_-VASc score was 3 and OACs were used in 57.1% of patients.

It will be important to consider the different populations when the medical outcomes in our intervention groups will be compared with those of the mentioned prior trials.

## 6. Limitations

Our sample has an underrepresentation of non-Caucasian AF patients compared to the composition of the current Flemish population. This is related to the “need to understand Dutch language” exclusion criterion for the AF-EduCare/AF-EduApp studies for which AF patients with an already documented other native language than Dutch (i.e., majority of non-Caucasian candidates), were not addressed and thus not registered in the eCRF. Secondly, all AF patients were recruited at tertiary/university centers in which all AF treatment possibilities were available. This may have led to a selection of AF patients with more complex medical (AF) histories and to a higher prevalence of rhythm control interventions than in the general population. Fourthly, a possible overestimation of paroxysmal AF and underestimation of persistent AF could be present. A correct AF classification often proved difficult due to a lack of documented temporal relationship in the medical files on the duration of the AF before an AF intervention. Finally, as data for the excluded patients were retrospectively collected, some parameters could not be retrieved or were uncertain.

## 7. Conclusions

The studied Belgian (Flemish) AF cohort is largely comparable with previous international registries regarding demographic and clinical cardiovascular profile. It shows, however, a higher use of anticoagulation and antiarrhythmic therapy (both drugs and interventions). Some AF risk factors likely are underrecognised (mainly OSA) and alcohol usage is reported to be higher (maybe due to more thorough questioning the patients about their lifestyle). In contrast to prior integrated AF trials, the AF-EduCare/AF-EduApp study incorporated all types of AF patients. Nevertheless, about a third of patients were not motivated enough to be included, pointing to the attention needed to attract all AF patients for active participation to integrated care. Follow-up and analysis of clinical outcomes of the study patients will provide further insights into the effectiveness and optimal modalities of integrated AF care.

## Data Availability

The raw data supporting the conclusions of this article will be made available by the authors upon request.
