# Supplementary material for: Baseline demographics of a contemporary Belgian atrial fibrillation cohort included in a large randomised clinical trial on targeted education and integrated care (AF-EduCare/AF-EduApp study)
Source: Front Cardiovasc Med. 2023 Jun 2;10:1186453. doi: 10.3389/fcvm.2023.1186453 (PMC10272799; doi:10.3389/fcvm.2023.1186453)
Supplement: Supplementary file 1 [file Table1.docx]

# Supplement

## **Supplementary table 1: Baseline characteristics of the on-treatment/Not eligible online study patients**

|  |  | Total  (n=347) | Online on-treatment (n=263) | Not Eligible (n=84) | P-value |
| --- | --- | --- | --- | --- | --- |
| Enrolment at outpatient clinic, n (%) |  | 163 (47.0) | 119 (45.2) | 44 (52.4) | 0.254 |
| AF-related, n (%) |  | 152 (93.3) | 111 (93.3) | 41 (93.2) | 1.000 |
| Unplanned, n (%) |  | 4 (2.5) | 3 (2.5) | 1 (2.3) | 1.000 |
| Enrolment at cardiology ward, n (%) |  | 184 (53.0) | 144 (54.8) | 40 (47.6) | 0.254 |
| AF-related, n (%) |  | 164 (89.1) | 128 (88.9) | 36 (90.0) | 1.000 |
| Unplanned, n (%) |  | 31 (16.8) | 25 (17.4) | 6 (15.0) | 0.724 |
| Highest level of education, n (%) |  |  |  |  | **<0.001** |
| Primary/Secondary school |  | 206 (59.4) | 138 (52.5) | 68 (81.0) |  |
| College/University |  | 141 (40.6) | 125 (47.5) | 16 (19.0) |  |
| Living alone, n (%) |  | 70 (20.2) | 49 (18.6) | 21 (25.0) | 0.205 |
| Internet accessibility, n (%) |  | 296 (85.3) | 263 (100.0) | 33 (39.3) | **<0.001** |
| Independent use, n (%) |  | 263 (88.9) | 263 (100.0) | 0 (0.0) | **<0.001** |
| In possession of: |  |  |  |  |  |
| PC/Laptop |  | 268 (77.2) | 245 (93.2) | 23 (27.4) | **<0.001** |
| Tablet |  | 152 (43.8) | 141 (53.6) | 11 (13.1) | **<0.001** |
| Smartphone |  | 196 (56.5) | 181 (68.8) | 15 (17.9) | **<0.001** |
| Treated by electrophysiologist |  | 201 (57.9) | 162 (61.6) | 39 (46.4) | **0.014** |
| Age (years), mean ± SD |  | 69.9 ± 9.2 | 68.3 ± 9.4 | 75.0 ± 6.2 | **<0.001** |
| Male, n (%) |  | 230 (66.3) | 179 (68.1) | 51 (60.7) | 0.215 |
| Belgian nationality, n (%) |  | 336 (96.8) | 253 (96.2) | 83 (98.8) | 0.472 |
| Race, n (%) |  |  |  |  | 1.000 |
| Caucasian, n (%) |  | 345 (99.4) | 261 (99.2) | 84 (100.0) |  |
| Other, n (%) |  | 2 (0.6) | 2 (0.8) | 0 (0.0) |  |
| BMI (kg/m^2^), mean ± SD |  | 28.0 ± 4.9 | 27.9 ± 5.0 | 28.2 ± 4.8 | 0.712 |
| Kind of AF, n (%) |  |  |  |  | 0.121 |
| First diagnosed |  | 49 (14.1) | 32 (12.2) | 17 (20.2) |  |
| Paroxysmal AF |  | 166 (47.8) | 129 (49.0) | 37 (44.0) |  |
| Persistent AF |  | 83 (23.9) | 69 (26.2) | 14 (16.7) |  |
| Long-standing persistent AF |  | 3 (0.9) | 2 (0.8) | 1 (1.2) |  |
| Permanent AF |  | 35 (10.1) | 24 (9.1) | 11 (13.1) |  |
| Atrial flutter |  | 11 (3.2) | 7 (2.7) | 4 (4.8) |  |
| Time since AF diagnosis (years), mean ± SD |  | 5.9 ± 7.5 | 5.6 ± 7.1 | 6.9 ± 8.7 | 0.100 |
| Rhythm at baseline, n (%) |  |  |  |  | 0.109 |
| Sinus rhythm |  | 260 (75.1) | 204 (77.9) | 56 (66.7) |  |
| AF/Atrial flutter |  | 81 (23.4) | 55 (21.0) | 26 (31.0) |  |
| Other rhythm |  | 5 (1.4) | 3 (1.1) | 2 (2.4) |  |
| CHA_2_DS_2_-VASc score, mean ± SD |  | 3.3 ± 1.7 | 2.9 ± 1.6 | 4.3 ± 1.6 | **<0.001** |
| HAS-BLED score, mean ± SD |  | 1.5 ± 0.9 | 1.4 ± 0.8 | 1.7 ± 0.9 | **0.005** |
| mEHRA, n (%) |  |  |  |  | 0.386 |
| 1 |  | 142 (40.9) | 105 (39.9) | 37 (44.0) |  |
| 2a |  | 104 (30.0) | 83 (31.6) | 21 (25.0) |  |
| 2b |  | 59 (17.0) | 45 (17.1) | 14 (16.7) |  |
| 3 |  | 37 (10.7) | 25 (9.5) | 12 (14.3) |  |
| 4 |  | 5 (1.4) | 5 (1.9) | 0 (0.0) |  |
| Concomitant disease, n (%) |  |  |  |  |  |
| (Coronary) artery disease |  | 111 (32.0) | 72 (27.4) | 39 (46.4) | **0.001** |
| History of congestive heart failure |  | 135 (38.9) | 93 (35.4) | 42 (50.0) | **0.017** |
| NYHA class III/IV |  | 27 (20.0) | 17 (18.3) | 10 (23.8) | 0.457 |
| Heart failure classification |  |  |  |  | 0.066 |
| HFpEF |  | 69 (51.1) | 43 (46.2) | 26 (61.9) |  |
| HFmrEF |  | 25 (18.5) | 16 (17.2) | 9 (21.4) |  |
| HFrEF |  | 41 (30.4) | 34 (36.6) | 7 (16.7) |  |
| Thyroid disease |  |  |  |  | 0.098 |
| Hyperthyroidism |  | 9 (2.6) | 4 (1.5) | 5 (6.0) |  |
| Hypothyroidism |  | 26 (7.5) | 20 (7.6) | 6 (7.1) |  |
| Severe kidney dysfunction° |  | 11 (3.2) | 8 (3.0) | 3 (3.6) | 0.731 |
| COPD |  | 20 (5.8) | 12 (4.6) | 8 (9.5) | 0.089 |
| Active malignancy |  | 15 (4.3) | 10 (3.8) | 5 (6.0) | 0.399 |
| Liver disease |  | 1 (0.3) | 0 (0.0) | 1 (1.2) | 0.242 |
| Cardiovascular risk factors, n (%) |  |  |  |  |  |
| Diabetes mellitus type I/II |  | 60 (17.3) | 44 (16.7) | 16 (19.0) | 0.625 |
| Hypertension |  | 226 (65.1) | 158 (60.1) | 68 (81.0) | **<0.001** |
| Hypercholesterolemia |  | 233 (67.1) | 175 (66.5) | 58 (69.0) | 0.670 |
| Current smoker |  | 33 (9.5) | 27 (10.3) | 6 (7.1) | 0.627 |
| Alcohol excess (≥ 8/day) |  | 66 (19.0) | 57 (21.7) | 9 (10.7) | **0.026** |
| Documented diagnosis of OSA* |  | 39 (11.5) | 30 (11.8) | 9 (10.8) | 0.819 |
| Physical inactivity (<60minutes/week)** |  | 171 (49.4) | 123 (46.8) | 48 (57.8) | 0.079 |
| Co-morbidities, n (%) |  |  |  |  |  |
| Previous TIA |  | 25 (7.2) | 15 (5.7) | 10 (11.9) | 0.056 |
| Previous ischaemic stroke |  | 21 (6.1) | 15 (5.7) | 6 (7.1) | 0.630 |
| Previous hemorrhagic stroke |  | 1 (0.3) | 1 (0.4) | 0 (0.0) | 1.000 |
| Other Ischaemic thrombo-embolic events |  | 3 (0.9) | 2 (0.8) | 1 (1.2) | 0.566 |
| History of pulmonary embolism |  | 5 (1.4) | 2 (0.8) | 3 (3.6) | 0.094 |
| Bleeding history |  | 9 (2.6) | 5 (1.9) | 4 (4.8) | 0.228 |
| Devices |  |  |  |  | 0.161 |
| PM |  | 25 (7.2) | 15 (5.7) | 10 (11.9) |  |
| ICD |  | 18 (5.2) | 15 (5.7) | 3 (3.6) |  |
| CRT-PM |  | 5 (1.4) | 5 (1.9) | 0 (0.0) |  |
| CRT-ICD |  | 8 (2.3) | 8 (3.0) | 0 (0.0) |  |
| Anticoagulation therapy, n (%) |  |  |  |  | **<0.001** |
| NOAC |  | 279 (80.4) | 206 (78.3) | 73 (86.9) |  |
| VKA |  | 25 (7.2) | 16 (6.1) | 9 (10.7) |  |
| LMWH |  | 1 (0.3) | 0 (0.0) | 1 (1.2) |  |
| None |  | 42 (12.1) | 41 (15.6) | 1 (1.2) |  |
| Combined anticoagulation/ antithrombotic therapy, n (%) |  |  |  |  |  |
| Triple therapy *(ASA+clopidogrel/ticagrelor+VKA/NOAC/ LMWH)* |  | 6 (1.7) | 3 (1.1) | 3 (3.6) | 0.155 |
| Dual therapy *(ASA/clopidogrel/ticagrelor+VKA/NOAC/ LMWH)* |  | 29 (8.4) | 18 (6.8) | 11 (13.1) | 0.071 |
| Dual antiplatelets *(ASA+clopidogrel/ticagrelor)* |  | 0 (0.0) | 0 (0.0) | 0 (0.0) | / |
| Only ASA |  | 12 (3.5) | 12 (4.6) | 0 (0.0) | 0.078 |
| Antiarrhythmic drugs, n (%) |  |  |  |  |  |
| Sotalol |  | 30 (8.6) | 25 (9.5) | 5 (6.0) | 0.313 |
| Flecainide |  | 67 (19.3) | 57 (21.7) | 10 (11.9) | **0.048** |
| Amiodarone |  | 56 (16.1) | 41 (15.6) | 15 (17.9 | 0.623 |
| Propafenone |  | 1 (0.3) | 0 (0.0) | 1 (1.2) | 0.242 |
| None |  | 197 (56.8) | 144 (54.8) | 53 (63.1) | 0.179 |
| Other drugs of interest, n (%) |  |  |  |  |  |
| Beta-Blockers |  | 246 (70.9) | 186 (70.7) | 60 (71.4) | 0.901 |
| Digoxin |  | 11 (3.2) | 10 (3.8) | 1 (1.2) | 0.472 |
| Non-DHP calcium-channel blockers |  | 12 (3.5) | 11 (4.2) | 1 (1.2) | 0.307 |
| ACE inhibitors |  | 135 (38.9) | 99 (37.6) | 36 (42.9) | 0.393 |
| ARBs |  | 72 (20.7) | 51 (19.4) | 21 (25.0) | 0.270 |
| Sacubitril/valsartan |  | 7 (2.0) | 7 (2.7) | 0 (0.0) | 0.202 |
| Thiazide diuretics |  | 76 (21.9) | 59 (22.4) | 17 (20.2) | **0.021** |
| Loop diuretics |  | 64 (18.4) | 42 (16.0) | 22 (26.2) | **0.035** |
| Aldosterone blockers |  | 69 (19.9) | 51 (19.4) | 18 (21.4) | 0.684 |
| Nitrates |  | 6 (1.7) | 5 (1.9) | 1 (1.2) | 1.000 |
| DHP calcium-channel blockers |  | 69 (19.9) | 50 (10.0) | 19 (22.6) | 0.471 |
| Central antihypertensive drugs |  | 7 (2.0) | 4 (1.5) | 3 (3.6) | 0.367 |
| Proton pomp inhibitors |  | 111 (32.0) | 81 (30.8) | 30 (35.7) | 0.400 |
| Oral antidiabetics |  | 45 (13.0) | 35 (13.3) | 10 (11.9) | 0.739 |
| Insulin |  | 13 (3.7) | 11 (4.2) | 2 (2.4) | 0.742 |
| Beta agonist |  | 24 (6.9) | 16 (6.1) | 8 (9.5) | 0.279 |
| Anticholinergic drugs |  | 17 (4.9) | 13 (4.9) | 4 (4.8) | 1.000 |
| Statins |  | 202 (58.2) | 151 (57.4) | 51 (60.7) | 0.593 |
| Hypolipidemic non-statin drugs |  | 27 (7.8) | 21 (8.0) | 6 (7.1) | 0.802 |
| Thyroid drugs |  | 23 (6.6) | 17 (6.5) | 6 (7.1) | 0.828 |
| NSAIDs |  | 5 (1.4) | 4 (1.5) | 1 (1.2) | 1.000 |
| AF Interventions, n (%) |  |  |  |  |  |
| Documented Pharmacological cardioversion |  | 63 (18.2) | 46 (17.5) | 17 (20.2) | 0.570 |
| Electrical cardioversion |  | 194 (55.9) | 147 (55.9) | 47 (56.0) | 0.992 |
| Catheter ablation |  | 108 (31.1) | 93 (35.3) | 15 (17.9) | **0.003** |
| Surgical therapy |  | 4 (1.2) | 3 (1.1) | 1 (1.2) | 1.000 |
| LAA closure device |  | 3 (0.9) | 2 (0.8) | 1 (1.2) | 0.566 |

AF: Atrial Fibrillation, SD: Standard Deviation, BMI: Body Mass Index, CHA_2_DS_2_-VASc: Congestive heart failure(1), Hypertension (1), Age ≥75 years (2), Diabetes mellitus (1), Stroke (2), Vascular disease (1), Age 65-74 years (1), Sex category (female=1); HAS-BLED: Systolic blood pressure >160mmHg (1), Abnormal renal and/or hepatic function (1 point each), Stroke (1), Bleeding history or predisposition (1), Labile INR (1), Age >65 years (1), Drugs or excessive alcohol drinking (1 point each), m: male, f: female, mEHRA: modified European Heart Rhythm Association classification, NYHA: New York Heart Association functional classification, HFpEF: Heart Failure with preserved Ejection Fraction, HFmrEF: Heart Failure with midrange Ejection Fraction, HFrEF: Heart Failure with reduced Ejection Fraction, COPD: Chronic Obstructive Pulmonary Disease, OSA: Obstructive Sleep Apnea, TIA: Transient Ischemic Attack, PM: Pacemaker, ICD: Implantable Cardioverter-Defibrillator, CRT: Cardiac Resynchronization Therapy, NOAC: Non-vitamin K antagonist Oral Anticoagulant, VKA: Vitamin K Antagonist, LMWH: Low-Molecular-Weight Heparins, ASA: Acetylsalicylic Acid, NSAIDs: Nonsteroidal Anti-Inflammatory Drugs, DHP: Dihydropyridine, ACE: Angiotensin Converting Enzyme, ARB: Angiotensin Receptor Blockers, LAA: Left Atrial Appendage.

° Dialysis, transplant, creatinine>2.26mg/d, * 9 unknown, **1 unknown

**Supplementary table 2: Baseline characteristics of the AF-EduApp study patients**

|  |  | Total (n=194) | Application on-treatment  (n=153) | Not Eligible  (n=41) | P-value |
| --- | --- | --- | --- | --- | --- |
| Enrolment at outpatient clinic, n (%) |  | 76 (39.2) | 58 (37.9) | 18 (43.9) | 0.485 |
| AF-related, n (%) |  | 69 (90.8) | 52 (89.7) | 17 (94.4) | 1.000 |
| Unplanned, n (%) |  | 1 (1.3) | 1 (1.7) | 0 (0.0) | 1.000 |
| Enrolment at cardiology ward, n (%) |  | 118 (60.8) | 95 (62.1) | 23 (56.1) | 0.485 |
| AF-related, n (%) |  | 102 (86.4) | 83 (87.4) | 19 (82.6) | 0.513 |
| Unplanned, n (%) |  | 30 (25.4) | 22 (23.2) | 8 (34.8) | 0.251 |
| Highest level of education, n (%) |  |  |  |  | **0.017** |
| Primary/Secondary school |  | 110 (56.7) | 80 (52.3) | 30 (73.2) |  |
| College/University |  | 84 (43.3) | 73 (47.7) | 11 (26.8) |  |
| Living alone, n (%) |  | 40 (20.6) | 30 (19.6) | 10 (24.4) | 0.501 |
| Internet accessibility, n (%) |  | 181 (93.3) | 153 (100.0) | 28 (68.3) | **<0.001** |
| Independent use, n (%) |  | 176 (97.2) | 153 (100.0) | 23 (82.1) | **<0.001** |
| In possession of: |  |  |  |  |  |
| PC/Laptop |  | 154 (79.4) | 132 (86.3) | 22 (53.7) | **<0.001** |
| Tablet |  | 105 (54.1) | 98 (64.1) | 7 (17.1) | **<0.001** |
| Smartphone |  | 156 (80.4) | 150 (98.0) | 6 (14.6) | **<0.001** |
| Treated by electrophysiologist |  | 86 (44.3) | 74 (48.4) | 12 (29.3) | **0.029** |
| Age (years), mean ± SD |  | 70.1 ± 7.0 | 68.8 ± 6.7 | 75.2 ± 5.6 | **<0.001** |
| Male, n (%) |  | 132 (68.0) | 105 (68.6) | 27 (65.9) | 0.735 |
| Belgian nationality, n (%) |  | 191 (98.5) | 150 (98.0) | 41 (100.0) | 1.000 |
| Race, n (%) |  |  |  |  | 1.000 |
| Caucasian, n (%) |  | 193 (99.5) | 152 (99.3) | 41 (100.0) |  |
| Other, n (%) |  | 1 (0.5) | 1 (0.7) | 0 (0.0) |  |
| BMI (kg/m^2^), mean ± SD |  | 28.5 ± 5.3 | 28.5 ± 5.2 | 28.3 ± 5.7 | 0.853 |
| Kind of AF, n (%) |  |  |  |  | **0.001** |
| First diagnosed |  | 30 (15.5) | 29 (19.0) | 1 (2.4) |  |
| Paroxysmal AF |  | 98 (50.5) | 78 (51.0) | 20 (48.8) |  |
| Persistent AF |  | 38 (19.6) | 31 (20.3) | 7 (17.1) |  |
| Long-standing persistent AF |  | 1 (0.5) | 1 (0.7) | 0 (0.0) |  |
| Permanent AF |  | 19 (9.8) | 8 (5.2) | 11 (26.8) |  |
| Atrial flutter |  | 8 (4.1) | 6 (3.9) | 2 (4.9) |  |
| Time since AF diagnosis (years), mean ± SD |  | 5.1 ± 6.4 | 5.2 ± 6.7 | 4.4 ± 5.3 | 0.463 |
| Rhythm at baseline, n (%) |  |  |  |  | 0.486 |
| Sinus rhythm |  | 140 (72.2) | 113 (73.9) | 27 (65.9) |  |
| AF/Atrial flutter |  | 53 (27.3) | 39 (25.5) | 14 (34.1) |  |
| Other rhythm |  | 1 (0.5) | 1 (0.7) | 0 (0.0) |  |
| CHA_2_DS_2_-VASc score, mean ± SD |  | 3.1 ± 1.6 | 2.9 ± 1.4 | 4.2 ± 1.7 | **<0.001** |
| HAS-BLED score, mean ± SD |  | 1.5 ± 0.9 | 1.6 ± 0.9 | 1.9 ± 1.0 | **0.041** |
| mEHRA, n (%) |  |  |  |  | 0.439 |
| 1 |  | 84 (43.3) | 65 (42.5) | 19 (46.3) |  |
| 2a |  | 61 (31.4) | 52 (34.0) | 9 (22.0) |  |
| 2b |  | 27 (13.9) | 21 (13.7) | 6 (14.6) |  |
| 3 |  | 21 (10.8) | 14 (9.2) | 7 (17.1) |  |
| 4 |  | 1 (0.5) | 1 (0.7) | 0 (0.0) |  |
| Concomitant disease, n (%) |  |  |  |  |  |
| (Coronary) artery disease |  | 62 (32.0) | 44 (28.8) | 18 (43.9) | 0.065 |
| History of congestive heart failure |  | 63 (32.5) | 45 (29.4) | 18 (43.9) | 0.078 |
| NYHA class III/IV |  | 16 (25.3) | 9 (20.0) | 7 (38.9) | 0.132 |
| Heart failure classification |  |  |  |  | 0.343 |
| HFpEF |  | 27 (42.9) | 17 (37.8) | 10 (55.6) |  |
| HFmrEF |  | 13 (20.6) | 9 (20.0) | 4 (22.2) |  |
| HFrEF |  | 23 (36.5) | 19 (42.2) | 4 (22.2) |  |
| Thyroid disease |  |  |  |  | 0.272 |
| Hyperthyroidism |  | 15 (7.7) | 14 (9.2) | 1 (2.4) |  |
| Hypothyroidism |  | 17 (8.8) | 12 (7.8) | 5 (12.2) |  |
| Severe kidney dysfunction° |  | 8 (4.1) | 6 (3.9) | 2 (4.9) | 0.677 |
| COPD |  | 16 (8.2) | 12 (7.8) | 4 (9.8) | 0.750 |
| Active malignancy |  | 5 (2.6) | 3 (2.0) | 2 (4.9) | 0.285 |
| Liver disease |  | 2 (1.0) | 2 (1.3) | 0 (0.0) | 1.000 |
| Cardiovascular risk factors, n (%) |  |  |  |  |  |
| Diabetes mellitus type I/II |  | 36 (18.6) | 24 (15.7) | 12 (29.3) | **0.047** |
| Hypertension |  | 125 (64.4) | 95 (62.1) | 30 (73.2) | 0.188 |
| Hypercholesterolemia* |  | 135 (69.9) | 99 (65.1) | 36 (87.8) | **0.005** |
| Current smoker |  | 20 (10.3) | 17 (11.1) | 3 (7.3) | 0.577 |
| Alcohol excess (≥ 8/day) |  | 59 (30.4) | 47 (30.7) | 12 (29.3) | 0.858 |
| Documented diagnosis of OSA** |  | 27 (14.1) | 23 (15.2) | 4 (9.8) | 0.456 |
| Physical inactivity (<60minutes/week) |  | 85 (43.8) | 62 (40.5) | 23 (56.1) | 0.074 |
| Co-morbidities, n (%) |  |  |  |  |  |
| Previous TIA |  | 15 (7.7) | 9 (5.9) | 6 (14.6) | 0.062 |
| Previous ischaemic stroke |  | 12 (6.2) | 8 (5.2) | 4 (9.8) | 0.284 |
| Previous hemorrhagic stroke |  | 1 (0.5) | 0 (0.0) | 1 (2.4) | 0.211 |
| Other Ischaemic thrombo-embolic events |  | 0 (0.0) | 0 (0.0) | 0 (0.0) | / |
| History of pulmonary embolism |  | 1 (0.5) | 1 (0.7) | 0 (0.0) | 1.000 |
| Bleeding history |  | 8 (4.1) | 5 (3.3) | 3 (7.3) | 0.369 |
| Devices |  |  |  |  | 0.281 |
| PM |  | 17 (8.8) | 16 (10.5) | 1 (2.4) |  |
| ICD |  | 6 (3.1) | 5 (3.3) | 1 (2.4) |  |
| CRT-PM |  | 2 (1.0) | 1 (0.7) | 1 (2.4) |  |
| CRT-ICD |  | 2 (1.0) | 1 (0.7) | 1 (2.4) |  |
| Anticoagulation therapy, n (%) |  |  |  |  | 0.337 |
| NOAC |  | 163 (86.0) | 126 (82.4) | 37 (90.2) |  |
| VKA |  | 16 (8.2) | 13 (8.5) | 3 (7.3) |  |
| LMWH |  | 0 (0.0) | 0 (0.0) | 0 (0.0) |  |
| None |  | 15 (7.7) | 14 (9.2) | 1 (2.4) |  |
| Combined anticoagulation/ antithrombotic therapy, n (%) |  |  |  |  |  |
| Triple therapy *(ASA+clopidogrel/ticagrelor+VKA/NOAC/ LMWH)* |  | 4 (2.1) | 4 (2.6) | 0 (0.0) | 0.581 |
| Dual therapy (ASA/clopidogrel/ticagrelor+VKA/NOAC/LMWH) |  | 22 (11.3) | 17 (11.1) | 5 (12.2) | 0.846 |
| Dual therapy *(ASA/clopidogrel/ticagrelor+VKA/NOAC/ LMWH)* |  | 0 (0.0) | 0 (0.0) | 0 (0.0) | / |
| Only ASA |  | 4 (2.1) | 3 (2.0) | 1 (2.4) | 1.000 |
| Antiarrhythmic drugs, n (%) |  |  |  |  |  |
| Sotalol |  | 12 (6.2) | 12 (7.8) | 0 (0.0) | 0.074 |
| Flecainide |  | 36 (18.6) | 32 (20.9) | 4 (9.8) | 0.118 |
| Amiodarone |  | 41 (21.1) | 33 (21.6) | 8 (19.5) | 0.775 |
| Propafenone |  | 1 (0.5) | 0 (0.0) | 1 (2.4) | 0.211 |
| None |  | 105 (54.1) | 77 (50.3) | 28 (68.3) | **0.040** |
| Other drugs of interest, n (%) |  |  |  |  |  |
| Beta-Blockers |  | 141 (72.7) | 113 (73.9) | 28 (68.3) | 0.478 |
| Digoxin |  | 3 (1.5) | 2 (1.3) | 1 (2.4) | 0.512 |
| Non-DHP calcium-channel blockers |  | 4 (2.1) | 4 (2.6) | 0 (0.0) | 0.581 |
| ACE inhibitors |  | 61 (31.4) | 45 (29.4) | 16 (39.0) | 0.239 |
| ARBs |  | 46 (23.7) | 35 (22.9) | 11 (26.8) | 0.597 |
| Sacubitril/valsartan |  | 5 (2.6) | 4 (2.6) | 1 (2.4) | 1.000 |
| Thiazide diuretics |  | 37 (19.1) | 32 (20.9) | 5 (12.2) | 0.207 |
| Loop diuretics |  | 39 (20.1) | 31 (20.3) | 8 (19.5) | 0.915 |
| Aldosterone blockers |  | 44 (22.7) | 33 (21.6) | 11 (26.8) | 0.475 |
| Nitrates |  | 5 (2.6) | 5 (3.3) | 0 (0.0) | 0.586 |
| DHP calcium-channel blockers |  | 42 (21.6) | 31 (20.3) | 11 (26.8) | 0.365 |
| Central antihypertensive drugs |  | 2 (1.0) | 2 (1.3) | 0 (0.0) | 1.000 |
| Proton pomp inhibitors |  | 70 (36.1) | 60 (39.2) | 10 (24.4) | 0.079 |
| Oral antidiabetics |  | 30 (15.5) | 20 (13.1) | 10 (24.4) | 0.075 |
| Insulin |  | 5 (2.6) | 4 (2.6) | 1 (2.4) | 1.000 |
| Beta agonist |  | 16 (8.2) | 12 (7.8) | 4 (9.8) | 0.750 |
| Anticholinergic drugs |  | 7 (3.6) | 4 (2.6) | 3 (7.3) | 0.165 |
| Statins |  | 106 (54.6) | 77 (50.3) | 29 (70.7) | **0.020** |
| Hypolipidemic non-statin drugs |  | 19 (9.8) | 14 (9.2) | 5 (12.2) | 0.560 |
| Thyroid drugs |  | 21 (10.8) | 16 (10.5) | 5 (12.2) | 0.750 |
| NSAIDs |  | 4 (2.1) | 4 (2.6) | 0 (0.0) | 0.581 |
| AF Interventions, n (%) |  |  |  |  |  |
| Documented Pharmacological cardioversion∀ |  | 29 (15.2) | 26 (17.2) | 3 (7.5) | 0.146 |
| Electrical cardioversion∀∀ |  | 111 (57.5) | 89 (58.2) | 22 (55.0) | 0.718 |
| Catheter ablation∀∀∀ |  | 70 (36.3) | 60 (39.2) | 10 (25.0) | 0.096 |
| Surgical therapy |  | 1 (0.5) | 0 (0.0) | 1 (2.4) | 0.211 |
| LAA closure device |  | 5 (2.6) | 2 (1.3) | 3 (7.3) | 0.064 |

AF: Atrial Fibrillation, SD: Standard Deviation, BMI: Body Mass Index, CHA_2_DS_2_-VASc: Congestive heart failure(1), Hypertension (1), Age ≥75 years (2), Diabetes mellitus (1), Stroke (2), Vascular disease (1), Age 65-74 years (1), Sex category (female=1); HAS-BLED: Systolic blood pressure >160mmHg (1), Abnormal renal and/or hepatic function (1 point each), Stroke (1), Bleeding history or predisposition (1), Labile INR (1), Age >65 years (1), Drugs or excessive alcohol drinking (1 point each), m: male, f: female, mEHRA: modified European Heart Rhythm Association classification, NYHA: New York Heart Association functional classification, HFpEF: Heart Failure with preserved Ejection Fraction, HFmrEF: Heart Failure with midrange Ejection Fraction, HFrEF: Heart Failure with reduced Ejection Fraction, COPD: Chronic Obstructive Pulmonary Disease, OSA: Obstructive Sleep Apnea, TIA: Transient Ischemic Attack, PM: Pacemaker, ICD: Implantable Cardioverter-Defibrillator, CRT: Cardiac Resynchronization Therapy, NOAC: Non-vitamin K antagonist Oral Anticoagulant, VKA: Vitamin K Antagonist, LMWH: Low-Molecular-Weight Heparins, ASA: Acetylsalicylic Acid, NSAIDs: Nonsteroidal Anti-Inflammatory Drugs, DHP: Dihydropyridine, ACE: Angiotensin Converting Enzyme, ARB: Angiotensin Receptor Blockers, LAA: Left Atrial Appendage.

° Dialysis, transplant, creatinine>2.26mg/d, * 1 unknown, **2 unknown,∀ 3 unknown,∀∀ 1 unknown,∀∀∀ 1 unknown

## **Supplementary table 3: Registries**

|  | **AF population registries** | | | | | | | **NOAC real-world registries** | | | |
| --- | --- | --- | --- | --- | --- | --- | --- | --- | --- | --- | --- |
|  | **ORBIT-AF I^1, 2^** | **EORP-AF**  **Pilot Registry^3^** | **PREFER in AF^4^** | **BALKAN-AF^5^** | **GARFIELD-AF cohort 4^1, 6^** | **ORBIT-AF II^1^** | **EORP-AF**  **Long-Term General Registry^7^** | **GLORIA-AF**  **(phase 2)^8^** | **Xantus^9^** | **APAF registry^10^** | **ETNA-AF-EU^11^** |
| Enrolment period | 2009-2011 | 2012-2013 | 2012-2013 | 2014-2015 | 2014-2015 | 2013-2016 | 2014-2016 | 2011-2014 | 2012-2013 | 2015-2017 | 2016-2018 |
| Countries | United States | 9 European Countries | 7 European Countries | 7 Balkan Countries | International | United States | 27 European Countries | International | International | Germany (multicenter) | 10 European countries |
| Design | Prospective | Prospective | Prospective | Prospective | Prospective | Prospective | Prospective | Prospective | Prospective | Prospective | Prospective |
| Specific inclusion remarks | Incident or prevalent AF | Qualifying AF episode <1 year | Prevalent AF | Incident or prevalent AF | New onset AF (≤6w) and ≥1  risk factor for  stroke | New onset AF (≤6m) or new on a NOAC (≤3m) | Qualifying AF episode <1 year | New onset AF (≤3m) and CHA_2_DS_2_-VASc ≥1 treated with VKA/dabigatran | Incident or prevalent AF, treated with rivaroxaban | Incident or prevalent AF | Qualifying AF episode <1 year, treated with edoxaban |
| Size (n) | 10132 | 3049 | 7243 | 2712 | 11046 | 11602 | 11096 | 15092 | 6785 | 5015 | 13980 |
| Inclusion at outpatient clinic (%) | 100.0 | 29.9 | / | / | / | 100.0 | 47.8 | / | 81.9 | / | / |
| AF related contact (%) | / | 60.5 | / | / | / | / | 65.8 | / | / | / | / |
| General demographics |  |  |  |  |  |  |  |  |  |  |  |
| Mean age (years) | 73.5 | 68.8 | 71.5 | 69.1 ± 10.9 | 69.6 ± 11.7 | 70.3 | 69.2 ± 11.4 | 70.5 ± 11.0 | 71.5 ± 10.0 | 74.2 | 73.6 ± 9.5 |
| Male (%) | 57.7 | 59.6 | 60.1 | 55.4 | 55.9 | 58.4 | 59.3 | 54.5 | 59.2 | 55.4 | 56.6 |
| Caucasian (%) | 89.2 | / | / | / | 63.8 | 85.5 | / | / | / | / | / |
| Mean BMI (kg/m2) | 30.5 | 28.0 | / | 27.7 ± 4.4 | / | 31.2 | / | 28.6 ± 6.3 | 28.3 ± 5.0 | / | 28.1 ± 5.1 |
| Current smoker (%) | 12.0 | 11 |  | 12.7 |  |  | 9.6 |  |  | / | 6.3 |
| Alcohol use (%)  Alcohol abuse (%) |  | 38.1  1.9 (>4/day) | 2.5 | 4.1 |  |  | 34.3  1.8 (≥4/day) | 6.6 |  | / | 55.2 |
| AF specifics |  |  |  |  |  |  |  |  |  |  |  |
| AF type (%):  -First diagnosis  -Paroxysmal  -Persistent  -Long-standing persistent  -Permanent  -Unknown | 5.0  50.0  16.8  /  27.9  / | 30.3  26.5  21.2  4.8  17.3  / | /  30.0  24.0  7.2  38.8 | 23.5  20.8  12.0  2.4  40.6  / |  |  | 15.6  25.7  19.1  4.3  33.5  1.7 | /  53.4  35.5  /  11.1  / | 18.5  40.6  13.6  /  27.0  0.2 | /  46.0  22.3  /  31.7  / | /  53.6  24.4  2.4  19.6  / |
| CHA_2_DS_2_-VASc (mean ± SD) | 3.9 ± 1.8 | 3.2 ± 1.8 | 3.4 | 3.5 ± 1.8 | 3 (median) | ≥ 2 = 85% | 3.1 ± 1.8 | 3.2 ± 1.5 | 3.4 ± 1.7 | 3.9 | 3.1 ± 1.4 |
| HAS-BLED (mean ± SD) |  | 1.4 ± 1.1 | 2.0 | 2.0 ± 1.2 | 1 (median) | 1-2=70.6% | 1.6 ± 1.1 | 1.4 ± 0.9 | 2.0 ± 1.0 | 2.3 | 2.6 ± 1.1 |
| Current AF symptoms (%) |  | 60.6 |  |  |  |  | 54.6 | 68.0 |  | / |  |
| AF interventions (%):  - Electrical  cardioversion  - Pharmacological cardioversion  - Catheter ablation | 30.1  5.5  / | 20.6  36.6  7.6 | 18.1  19.5  5.0 |  |  |  | 19.2  23.0  6.9 | 16.1  1.1 |  | 28.3  /  8.1 |  |
| Concomitant diseases |  |  |  |  |  |  |  |  |  |  |  |
| Hypertension (%) | 83.0 | 70.9 | 72.0 | 79.2 | 74.7 | 79.6 | 62.1 | 74.6 | 74.7 | 86.4 | 76.9 |
| Congestive heart failure (%)  NYHA III/IV (%) | 32.5    22.2 | 47.5    41.2 | 21.3 | 43.5 | 19.3 | 21.0    18.4 | 39.5    35.9 | 24.2 | 18.6 | 37.9 | 5.8 |
| Diabetes mellitus (%) | 29.4 | 20.6 | 22.4 | 25.0 | 22.1 | 26.2 | 23.0 | 23.1 | 19.6 | 29.8 | 21.9 |
| Coronary artery disease (%) | 36.0 | 36.4 | 23.4 | 30.7 | 22.0 | 26.6 | 29.1 | 20.3 | 10.1 (MI) | 33.9 |  |
| Hyperthyroidism (%) |  | 3.0 |  | / | / | / | 4.6 | / | / | / | / |
| Hypothyroidism |  | 7.2 |  | / | / | / | 9.5 | / | / | / | / |
| Hypercholesterolemia (%) |  | 48.6 |  | / | / | / | 41.4 | 39.9 | / | / | / |
| COPD (%) | 16.4 | 11.1 |  | / | / | / | 8.9 | / | / | / | / |
| Previous TIA (%) | 15.1 | 4.1 |  | 3.1 | 11.0 | 10.8 | 3.0 | 14.2 | 19.0 | 4.9 | 3.3 |
| Previous stroke (%) |  | 6.4 | 8.4 | 10.5 |  |  | 6.2 |  |  | 11.1 | 5.9 |
| Other trombo-embolic events (%) |  | 13.1 |  | / |  |  | 11.6 |  |  | / |  |
| Bleeding events (%) |  | 5.9 | 7.3 | 5.0 | 2.4 |  | 5.2 | 5.6 |  | 2.2 | 3.1 |
| Malignancy (%) |  | 5.3 |  | / |  |  | 7.4 | 9.3 |  | / |  |
| Chronic kidney disease (%) | 1.3 (only dialysis) | 13.2 | 1.7 (CrCl <30mL/min) | 15.5 |  |  | 12.5 | 1.6 | 1.4 (CrCl <30mL/min) | / |  |
| Drug therapy |  |  |  |  |  |  |  |  |  |  |  |
| VKA (%) | 71.3 | 71.6 | 78.0 | 60.9 |  | 17 | 50.2 | 32.2 |  | 31.6 | / |
| NOAC (%) | 4.9 | 8.4 | 6.1 | 12.7 | / | 56 | 34.8 | 47.6 | 100.0 | 63.6 | 100.0 |
| No antitrombotics (%) | 5.1 | 4.8 | 6.5 | 9.9 |  |  | 6.4 | 7.8 |  | 3.7 |  |
| Antiarrhythmic drugs (%) | 28.7 | 36.0 | 47.3 |  |  |  | 27.8 |  |  | / |  |
| ACE inhibitors (%) | / | 43.1 |  |  |  |  | 42.9 |  |  | / |  |
| Beta-blockers (%) | 63.9 | 69.2 |  |  |  |  | 69.2 |  |  | / |  |
| Non-DHP calcium-channel blockers (%) | / | 6.2 |  |  |  |  | / |  |  | / |  |
| Digoxin (%) | 23.4 | 19.4 |  |  |  |  | 14.7 |  |  | / |  |
| Diuretics (%) | / | 50.8 |  |  |  |  | 51.6 |  |  | / |  |
| Statins (%) | / | 49.4 |  |  |  |  | 42.2 |  |  | / |  |

AF: Atrial Fibrillation, BMI: Body Mass Index, CHA_2_DS_2_-VASc: Congestive heart failure(1), Hypertension (1), Age ≥75 years (2), Diabetes mellitus (1), Stroke (2), Vascular disease (1), Age 65-74 years (1), Sex category (female=1); HAS-BLED: Systolic blood pressure >160mmHg (1), Abnormal renal and/or hepatic function (1 point each), Stroke (1), Bleeding history or predisposition (1), Labile INR (1), Age >65 years (1), Drugs or excessive alcohol drinking (1 point each), NYHA: New York Heart Association functional classification, COPD: Chronic Obstructive Pulmonary Disease, TIA: Transient Ischemic Attack, VKA: Vitamin K Antagonist, NOAC: Non-vitamin K antagonist Oral Anticoagulant, ACE: Angiotensin Converting Enzyme, DHP: Dihydropyridine,‘Grey boxes’ indicate the registries including only early onset AF patients

## **Supplementary table 4: Integrated AF care studies**

|  | **Hendriks et al.^12^** | **Stewart et al.**  (SAFETY)^13^ | **Carter et al.^14^** | **Wijtvliet et al.** (RACE 4)^15^ | **Van den Dries et al.** (ALL-IN)^16^ | **Cox et al.** (IMPACT-AF)^17^ | **Guo et al.** (mAFA-II trial)^18, 19^ | **Hendriks / Sanders** (HELP-AF) |
| --- | --- | --- | --- | --- | --- | --- | --- | --- |
| Year of publication | 2012 | 2015 | 2016 | 2019 | 2020 | 2020 | 2020 | Not published yet |
| Country | the Netherlands | Australia | Canada | the Netherlands | the Netherlands | Canada | China | Australia |
| Study type | Single-center RCT | Multicenter RCT | Multicenter before & after study | Multicenter RCT | Multicenter RCT (clusters) | Multicenter RCT (clusters) | Multicenter RCT  (clusters) | Multicenter RCT |
| Size (n) | 712 | 335 | 413 | 1354 | 1240 | 1133 | 2473 | 627 |
| Recruitment specifics | Outpatient clinic | Admitted, known with chronic AF but no heart failure | Emergency department with new AF diagnosis | Outpatient clinic  with new AF diagnosis (<3 months) or history of AF with no regular AF control (<24 months) | Primary care, documented AF and ≥ 65 years | Primary care | Outpatient and inpatient departments and with CHA_2_DS_2_-VASc score ≥ 2 | Emergency department with a primary AF diagnosis |
| Intervention | Nurse-led outpatient care | Nurse-led care (home visits) | Nurse-led outpatient care | Nurse-led outpatient care | Nurse-led  outpatient care | Clinical decision support system supporting GPs | Mobile Application and clinical decision support tools | Home-based intervention |
| Mean follow-up | 22 months | Minimum of 24 months | Minimum of 12 months | 37 months | 24 months | Minimum of 12 months | 37.4 weeks vs 41.6 weeks | 24 months |
| Primairy endpoint | CV death and hospitalisation | All-cause mortality and/or unplanned  readmission | Death, CV hospitalization, AF ED visit | CV death and hospitalisations | All-cause mortality | Unplanned CV hospitalization and AF ED visit | Stroke/ thromboembolism,  all-cause death, CV rehospitalization | Total unplanned hospital admissions |
| Result (intervention vs control group) | 14.3% vs 20.8%  (p=0.029) | 75.6% vs 81.5% (p=0.851) | 18.4% vs 28.5% (p=0.017)  Matched cohorts: 17.3% vs 26.2% (p=0.049) | 9.7% vs 11.6% /year (p=0.12) | 7.4% vs 13.5% RR=0.55 (0.37-0.82, p=0.003) | 12.7% vs 13.4% (p=0.713) | 1.9% vs 6.0% (p<0.001), FU <1 year  6.9% vs 13.6% (p<0.001), FU >1 year | Reduced unplanned hospitalizations:  a. Total -26%  b. AF-related -31%  c. Other cardiac -49% |
| General demography |  |  |  |  |  |  |  |  |
| Mean age (years) | 66.5 ± 13 | 72 ± 11 | 63.8 ± 14.7 | 64.0 ± 10.5 | 77 (median) | 72.3 ± 10.0 | 68.5 ± 13.6 | 70 (median) |
| Male (%) | 58.7 | 51.9 | 57.9 | 65.8 | 50.6 | 61.9 | 61.9 | 55.8 |
| Caucasian (%) | / | / | / | / | / | / | / | / |
| BMI (kg/m2) | 27.2 ± 5.1 | 29 (median) | / | 28.0 ± 5.0 | / | / | / | / |
| Current smoker (%) | / | 13.5 | / | 9.3 | / | / | 9.8 | / |
| Alcohol use (%)  Alcohol excess | / | /  16.0 | / | / | / | / | / | / |
| AF specifics |  |  |  |  |  |  |  |  |
| Type of AF (%):  First diagnosed  Paroxysmal  Persistent  Long-standing  persistent  Permanent  Unknown | /  55.2  15.7  /  22.3  / | 23.2 (Newly diagnosed)  /  90.1  /  /  / | 100.0  /  /  /  /  / | /  62.0  17.2  /  5.4  14.8 | /  /  /  /  /  / | /  /  /  /  /  / | 12.8  40.1  24.9  4.7  5.1  11.9 | 100.0  /  /  /  /  / |
| CHA_2_DS_2_-VASc (mean ± SD) | 2.4 ± 1.7 | 3.6 ± 1.8 | 2.2 ± 1.8 | ≥ 2 = 56.6% | / | 3.7 ± 1.8 | 3 (median) | 2.7 ± 1.9 |
| HAS-BLED (mean ± SD) | / | / | / | ≥ 3 = 7.8% | / | / | 1 (median) | / |
| Current AF symptoms (%) | 82.6 | 82.9 | / | 45.1 | / | / | / | / |
| Concomitant diseases |  |  |  |  |  |  |  |  |
| Hypertension (%) | 53.4 | 71.6 | 35.4 | 47.6 | 56.5 | 78.6 | 56.3 | 61.4 |
| Congestive heart failure (%)  NYHA III/IV (%) | 7.0  / | 0.0  / | 9.9  / | 11.7  / | 16.8  / | 25.7  / | 21.5  / | 9.8  / |
| Diabetes mellitus (%) | 13.5 | 28.7 | 10.9 | 9.7 | 25.5 | 28.9 | 22.5 | 14.4 |
| Coronary artery disease (%) | 10.0 | 33.4 | 9.2 | 5.7 | 17.2 | 35.0 | 40.9 | / |
| Hyperthyroidism (%) | 3.4 | / | / | 1.5 | / | / | 2.6 | / |
| Hypothyroidism | / | / | / | / | / | / | / | / |
| Hypercholesterolaemia (%) | / | 55.0 | / | / | / | / | / | 53.6 |
| COPD (%) | 8.4 | 16.1 (Chronic airway limitation) | 11.4 | 9.2 | 13.9 | / | / | / |
| Previous TIA (%) | 12.5 | 15.5 | 6.8 | 7.2 | 14.4 | 18.4 | / | 11.2 |
| Previous stroke (%) |  |  |  |  |  |  | 12.7 |  |
| Other trombo-embolic events (%) | / | / | / | / | 4.4 |  | 3.4 | / |
| Bleeding events (%) | / | / | / | / | / | / | / | / |
| Malignancy (%) | / | / | / | 9.3 | 18.2 | / | / | / |
| Chronic kidney disease (%) | / | 35.1 (eGFR <60 mL/min/1.73m²) | / | / | 13.6 | / | 9.3 | / |
| OSA | / | / | 7.0 | / | / | / | / | 13.6 |
| PM | / | 9.0 | 2.2 | / | 7.7 | / | / | / |
| Drug therapy |  |  |  |  |  |  |  |  |
| VKA (%) | 57.0 | 55.5 | 17.4 | 33.6 | 77.5 | 42.0 | 5.9 | / |
| ASA (%) | 31.7 | 48.0 | 49.2 | 8.1 | / | / | 12.2 | / |
| NOAC (%) | / | / | 13.6 | 34.0 | 13.2 | 29.3 | 51.2 | / |
| No antitrombotics (%) | / | / | 69.0 | / | 9.3 | 15.6 | 24.2 | / |
| Antiarrhythmic drugs (%) | 27.1 | 86.6 (at discharge) | 2.7 | 14.4 | 6.8 | / | 10.7 | / |
| Beta-blockers (%) | 49.3 | 49.3 | 52.2 | 58.1 | 72.6 | / | 25.3 | / |
| Digoxin (%) | 14.2 | 34.9 | 2.2 | 6.7 | 18.9 | / | 5.4 | / |
| Diuretics (%) | 17.3 | 41.5 | 28.3 | 20.9 | 43.5 | / | 15.3 | / |
| Statins (%) | 30.6 | / | / | 24.1 | / | / | 35.1 | / |

RCT: Randomized Clinical Trial, AF: Atrial Fibrillation, CV: Cardiovascular, FU: Follow-Up, BMI: Body Mass Index, CHA_2_DS_2_-VASc: Congestive heart failure(1), Hypertension (1), Age ≥75 years (2), Diabetes mellitus (1), Stroke (2), Vascular disease (1), Age 65-74 years (1), Sex category (female=1); HAS-BLED: Systolic blood pressure >160mmHg (1), Abnormal renal and/or hepatic function (1 point each), Stroke (1), Bleeding history or predisposition (1), Labile INR (1), Age >65 years (1), Drugs or excessive alcohol drinking (1 point each), NYHA: New York Heart Association functional classification, COPD: Chronic Obstructive Pulmonary Disease, TIA: Transient Ischemic Attack, OSA: Obstructive Sleep Apnea, PM: Pacemaker, VKA: Vitamin K Antagonist, ASA: Acetylsalicylic Acid, NOAC: Non-vitamin K antagonist Oral Anticoagulant

## **References**

1. Steinberg BA, Gao H, Shrader P, Pieper K, Thomas L, Camm AJ, Ezekowitz MD, Fonarow GC, Gersh BJ, Goldhaber S, Haas S, Hacke W, Kowey PR, Ansell J, Mahaffey KW, Naccarelli G, Reiffel JA, Turpie A, Verheugt F, Piccini JP, Kakkar A, Peterson ED, Fox KAA, Garfield AF and Investigators O-A. International trends in clinical characteristics and oral anticoagulation treatment for patients with atrial fibrillation: Results from the GARFIELD-AF, ORBIT-AF I, and ORBIT-AF II registries. *Am Heart J* 2017; 194: 132-140. 2017/12/11. DOI: 10.1016/j.ahj.2017.08.011.

2. Fosbol EL, Holmes DN, Piccini JP, Thomas L, Reiffel JA, Mills RM, Kowey P, Mahaffey K, Gersh BJ, Peterson ED, Investigators O-A and Patients. Provider specialty and atrial fibrillation treatment strategies in United States community practice: findings from the ORBIT-AF registry. *J Am Heart Assoc* 2013; 2: e000110. 2013/07/23. DOI: 10.1161/JAHA.113.000110.

3. Lip GY, Laroche C, Dan GA, Santini M, Kalarus Z, Rasmussen LH, Oliveira MM, Mairesse G, Crijns HJ, Simantirakis E, Atar D, Kirchhof P, Vardas P, Tavazzi L and Maggioni AP. A prospective survey in European Society of Cardiology member countries of atrial fibrillation management: baseline results of EURObservational Research Programme Atrial Fibrillation (EORP-AF) Pilot General Registry. *Europace* 2014; 16: 308-319. 2013/12/20. DOI: 10.1093/europace/eut373.

4. Kirchhof P, Ammentorp B, Darius H, De Caterina R, Le Heuzey JY, Schilling RJ, Schmitt J and Zamorano JL. Management of atrial fibrillation in seven European countries after the publication of the 2010 ESC Guidelines on atrial fibrillation: primary results of the PREvention oF thromboemolic events--European Registry in Atrial Fibrillation (PREFER in AF). *Europace* 2014; 16: 6-14. 2013/10/03. DOI: 10.1093/europace/eut263.

5. Potpara TS, Dan GA, Trendafilova E, Goda A, Kusljugic Z, Manola S, Music L, Musetescu R, Badila E, Mitic G, Paparisto V, Dimitrova ES, Polovina MM, Petranov SL, Djergo H, Loncar D, Bijedic A, Brusich S, Lip GY and Investigators B-A. Stroke prevention in atrial fibrillation and 'real world' adherence to guidelines in the Balkan Region: The BALKAN-AF Survey. *Sci Rep* 2016; 6: 20432. 2016/02/13. DOI: 10.1038/srep20432.

6. Camm AJ, Accetta G, Ambrosio G, Atar D, Bassand JP, Berge E, Cools F, Fitzmaurice DA, Goldhaber SZ, Goto S, Haas S, Kayani G, Koretsune Y, Mantovani LG, Misselwitz F, Oh S, Turpie AG, Verheugt FW, Kakkar AK and Investigators G-A. Evolving antithrombotic treatment patterns for patients with newly diagnosed atrial fibrillation. *Heart* 2017; 103: 307-314. 2016/09/21. DOI: 10.1136/heartjnl-2016-309832.

7. Boriani G, Proietti M, Laroche C, Fauchier L, Marin F, Nabauer M, Potpara T, Dan GA, Kalarus Z, Diemberger I, Tavazzi L, Maggioni AP, Lip GYH, Investigators E-AL-TGR and Steering C. Contemporary stroke prevention strategies in 11 096 European patients with atrial fibrillation: a report from the EURObservational Research Programme on Atrial Fibrillation (EORP-AF) Long-Term General Registry. *Europace* 2018; 20: 747-757. 2017/10/11. DOI: 10.1093/europace/eux301.

8. Mazurek M, Halperin JL, Huisman MV, Diener HC, Dubner SJ, Ma CS, Rothman KJ, Healey JS, Teutsch C, Paquette M, Franca LR, Lu S, Bartels DB and Lip GYH. Antithrombotic treatment for newly diagnosed atrial fibrillation in relation to patient age: the GLORIA-AF registry programme. *Europace* 2020; 22: 47-57. 2019/10/28. DOI: 10.1093/europace/euz278.

9. Camm AJ, Amarenco P, Haas S, Hess S, Kirchhof P, Kuhls S, van Eickels M, Turpie AG and Investigators X. XANTUS: a real-world, prospective, observational study of patients treated with rivaroxaban for stroke prevention in atrial fibrillation. *Eur Heart J* 2016; 37: 1145-1153. 2015/09/04. DOI: 10.1093/eurheartj/ehv466.

10. Zeymer U, Lober C, Wolf A, Richard F, Schafer H, Taggeselle J, Kabitz HJ, Prondzinsky R, Suselbeck T and Investigators A. Use, Persistence, Efficacy, and Safety of Apixaban in Patients with Non-Valvular Atrial Fibrillation in Unselected Patients in Germany. Results of the Prospective Apixaban in Atrial Fibrillation (APAF) Registry. *Cardiol Ther* 2020; 9: 467-478. 2020/07/09. DOI: 10.1007/s40119-020-00188-1.

11. De Caterina R, Kelly P, Monteiro P, Deharo JC, de Asmundis C, Lopez-de-Sa E, Weiss TW, Waltenberger J, Steffel J, de Groot JR, Levy P, Bakhai A, Zierhut W, Laeis P, Kerschnitzki M, Reimitz PE, Kirchhof P and investigators ET-A-E. Characteristics of patients initiated on edoxaban in Europe: baseline data from edoxaban treatment in routine clinical practice for patients with atrial fibrillation (AF) in Europe (ETNA-AF-Europe). *BMC Cardiovasc Disord* 2019; 19: 165. 2019/07/14. DOI: 10.1186/s12872-019-1144-x.

12. Hendriks JM, de Wit R, Crijns HJ, Vrijhoef HJ, Prins MH, Pisters R, Pison LA, Blaauw Y and Tieleman RG. Nurse-led care vs. usual care for patients with atrial fibrillation: results of a randomized trial of integrated chronic care vs. routine clinical care in ambulatory patients with atrial fibrillation. *Eur Heart J* 2012; 33: 2692-2699. 2012/03/29. DOI: 10.1093/eurheartj/ehs071.

13. Stewart S, Ball J, Horowitz JD, Marwick TH, Mahadevan G, Wong C, Abhayaratna WP, Chan YK, Esterman A, Thompson DR, Scuffham PA and Carrington MJ. Standard versus atrial fibrillation-specific management strategy (SAFETY) to reduce recurrent admission and prolong survival: pragmatic, multicentre, randomised controlled trial. *Lancet* 2015; 385: 775-784. 2014/12/04. DOI: 10.1016/S0140-6736(14)61992-9.

14. Carter L, Gardner M, Magee K, Fearon A, Morgulis I, Doucette S, Sapp JL, Gray C, Abdelwahab A and Parkash R. An Integrated Management Approach to Atrial Fibrillation. *J Am Heart Assoc* 2016; 5 2016/01/27. DOI: 10.1161/JAHA.115.002950.

15. Wijtvliet E, Tieleman RG, van Gelder IC, Pluymaekers N, Rienstra M, Folkeringa RJ, Bronzwaer P, Elvan A, Elders J, Tukkie R, Luermans J, Van Asselt A, Van Kuijk SMJ, Tijssen JG, Crijns H and Investigators R. Nurse-led vs. usual-care for atrial fibrillation. *Eur Heart J* 2020; 41: 634-641. 2019/09/24. DOI: 10.1093/eurheartj/ehz666.

16. van den Dries CJ, van Doorn S, Rutten FH, Oudega R, van de Leur S, Elvan A, Oude Grave L, Bilo HJG, Moons KGM, Hoes AW and Geersing GJ. Integrated management of atrial fibrillation in primary care: results of the ALL-IN cluster randomized trial. *Eur Heart J* 2020; 41: 2836-2844. 2020/03/01. DOI: 10.1093/eurheartj/ehaa055.

17. Cox JL, Parkash R, Foster GA, Xie F, MacKillop JH, Ciaccia A, Choudhri SH, Hamilton LM, Nemis-White JM, Thabane L and Investigators I-A. Integrated Management Program Advancing Community Treatment of Atrial Fibrillation (IMPACT-AF): A cluster randomized trial of a computerized clinical decision support tool. *Am Heart J* 2020; 224: 35-46. 2020/04/18. DOI: 10.1016/j.ahj.2020.02.019.

18. Guo Y, Lane DA, Wang L, Zhang H, Wang H, Zhang W, Wen J, Xing Y, Wu F, Xia Y, Liu T, Wu F, Liang Z, Liu F, Zhao Y, Li R, Li X, Zhang L, Guo J, Burnside G, Chen Y, Lip GYH and m AFAIITI. Mobile Health Technology to Improve Care for Patients With Atrial Fibrillation. *J Am Coll Cardiol* 2020; 75: 1523-1534. 2020/04/04. DOI: 10.1016/j.jacc.2020.01.052.

19. Guo Y, Guo J, Shi X, Yao Y, Sun Y, Xia Y, Yu B, Liu T, Chen Y, Lip GYH and m AFAIITi. Mobile health technology-supported atrial fibrillation screening and integrated care: A report from the mAFA-II trial Long-term Extension Cohort. *Eur J Intern Med* 2020; 82: 105-111. 2020/10/18. DOI: 10.1016/j.ejim.2020.09.024.
